# Development, validation, and proof-of-concept implementation of a two-year risk prediction model for undiagnosed atrial fibrillation using common electronic health data (UNAFIED)

**DOI:** 10.1186/s12911-021-01482-1

**Published:** 2021-04-03

**Authors:** Randall W. Grout, Siu L. Hui, Timothy D. Imler, Sarah El-Azab, Jarod Baker, George H. Sands, Mohammad Ateya, Francis Pike

**Affiliations:** 1 Center for Biomedical Informatics, Regenstrief Institute, 1101 W Tenth St, Indianapolis, IN 46202 USA; 2 Department of Pediatrics, Indiana University School of Medicine, Indianapolis, IN USA; 3 Research Services, Regenstrief Institute, Indianapolis, IN USA; 4 Department of Biostatistics, Indiana University School of Medicine, Indianapolis, IN USA; 5 Division of Gastroenterology and Hepatology, Indiana University School of Medicine, Indianapolis, IN USA; 6 Pfizer Inc, US Medical Affairs, New York, NY USA

**Keywords:** Atrial fibrillation, Screening, Machine learning, Predictive model, Electronic health record, Decision support

## Abstract

**Background:**

Many patients with atrial fibrillation (AF) remain undiagnosed despite availability of interventions to reduce stroke risk. Predictive models to date are limited by data requirements and theoretical usage. We aimed to develop a model for predicting the 2-year probability of AF diagnosis and implement it as proof-of-concept (POC) in a production electronic health record (EHR).

**Methods:**

We used a nested case–control design using data from the Indiana Network for Patient Care. The development cohort came from 2016 to 2017 (outcome period) and 2014 to 2015 (baseline). A separate validation cohort used outcome and baseline periods shifted 2 years before respective development cohort times. Machine learning approaches were used to build predictive model. Patients ≥ 18 years, later restricted to age ≥ 40 years, with at least two encounters and no AF during baseline, were included. In the 6-week EHR prospective pilot, the model was silently implemented in the production system at a large safety-net urban hospital. Three new and two previous logistic regression models were evaluated using receiver-operating characteristics. Number, characteristics, and CHA_2_DS_2_-VASc scores of patients identified by the model in the pilot are presented.

**Results:**

After restricting age to ≥ 40 years, 31,474 AF cases (mean age, 71.5 years; female 49%) and 22,078 controls (mean age, 59.5 years; female 61%) comprised the development cohort. A 10-variable model using age, acute heart disease, albumin, body mass index, chronic obstructive pulmonary disease, gender, heart failure, insurance, kidney disease, and shock yielded the best performance (C-statistic, 0.80 [95% CI 0.79–0.80]). The model performed well in the validation cohort (C-statistic, 0.81 [95% CI 0.8–0.81]). In the EHR pilot, 7916/22,272 (35.5%; mean age, 66 years; female 50%) were identified as higher risk for AF; 5582 (70%) had CHA_2_DS_2_-VASc score ≥ 2.

**Conclusions:**

Using variables commonly available in the EHR, we created a predictive model to identify 2-year risk of developing AF in those previously without diagnosed AF. Successful POC implementation of the model in an EHR provided a practical strategy to identify patients who may benefit from interventions to reduce their stroke risk.

**Supplementary Information:**

The online version contains supplementary material available at 10.1186/s12911-021-01482-1.

## Background

Atrial fibrillation (AF) is associated with increased risk of stroke or systemic embolization leading to significant patient morbidity and mortality [1]. Diagnosed AF was estimated to affect up to approximately 8.7 million people in the United States in 2021 [2, 3]. A recent study estimated the undiagnosed AF prevalence in the United States at 1.3% (95% CI 0.9–1.9%) in those over age 65 [4]. Among undiagnosed cases, 56% had a CHADS2 score ≥ 2 (moderate to high risk of stroke) with a substantial potential for risk-reduction with guideline-recommended anticoagulation [3].

Multiple studies sought to develop predictive risk models for AF in an undiagnosed population. The Framingham Heart Study (FHS) AF Risk Score predicted 10-year AF [5]. The Atherosclerosis Risk in Communities (ARIC) study provided an alternative AF risk score in a different patient population [6]. Both FHS and ARIC models were derived from single-community cohorts. The CHARGE-AF model combined three cohorts from different studies and provided a 5-year AF risk simple model mostly using variables commonly collected in primary care and a more complex model using ECG variables [7]. Other efforts to identify machine learning models have looked at hundreds of variables [8].

However, these existing models required variables not typically available as structured electronic health record (EHR) fields (e.g., electrocardiogram [ECG] parameters). Newer attempts at EHR-based models have required extra steps of data harmonization [9] or use of long prediction horizons and require non-missing data or oft-unstructured data [10]. Other efforts use available data from the EHR, but make a prediction for 10-year risk using binned risk categories [11], or rely on claims databases and make predictions on simple cross-sectional association [12]. A 5 to 10-year AF risk may limit a meaningful patient screening intervention compared to a relatively imminent risk in a 2-year horizon.

A prospective AF-risk model implemented in the EHR to help find patients at higher risk of undiagnosed AF, with potential diagnosis and guideline-recommended treatment, may reduce their risk of thrombosis (stroke or systemic embolism) and be welcomed by patients and their families. Clinicians and health systems may also find this system useful in population health management. We aimed to develop and validate a model using EHR data from multiple health systems in a regional health information exchange (HIE) to estimate 2-year risk of AF diagnosis. Our second goal was to assess the feasibility of implementing this model in a real EHR within a single health system.

## Methods

### Study setting

The predictive model was developed and validated with data collected from the Indiana Network for Patient Care (INPC) HIE. The INPC encompasses clinical data from over 100 healthcare entities in and around Indiana, including hospitals, health networks, and insurance providers, collected over more than 30 years from more than 18 million patients.. The proof-of-concept implementation was performed at Eskenazi Health, a 315-bed safety-net hospital with 10 federally-qualified community health centers for Marion County in central Indiana (including Indianapolis). It hosts nearly one million visits per year among the health centers, hospital, emergency department, and mental health system. For patients with hospital and office encounters in 2019, they self-identified as 26% Hispanic/Latino, 55% female, 42% Black, and 31% White.

### Compliance and data sources

This study was approved by the Indiana University Institutional Review Board and Regenstrief Institute Data Management Committee. A waiver of informed consent was granted by the Indiana University Institutional Review Board. The methods were carried out in accordance with relevant guidelines and regulations. We queried EHR data gathered between 1/1/2014 and 12/31/2017 from the INPC, which includes Eskenazi Health. The reporting of the study followed the Strengthening the Reporting of Observational Studies in Epidemiology (STROBE) guidelines and adhered to the Transparent Reporting of a Multivariable Prediction Model for Individual Prognosis or Diagnosis (TRIPOD) recommendations (see Additional file 1 for TRIPOD statement) [13, 14].

### Study design

The model was developed on observational data from a retrospective cohort using a nested case–control design. The primary outcome of AF identification was observed from 1/1/2016 to 12/31/2017 (outcome period) following a baseline period from 1/1/2014 to 12/31/2015 for extracting candidate prediction variables. A validation cohort was developed with inclusion/exclusion criteria identical to the development cohort, with observation periods shifted 2 years earlier relative to the development cohort (i.e., 1/1/2014–12/31/2015 for outcome period, 1/1/2012–12/31/2013 for baseline period). The final model was implemented into Eskenazi Health’s Epic EHR for a 6-week proof-of-concept study.

### Identification of cases

AF was determined if the patients had an International Statistical Classification of Diseases and Related Health Problems (ICD) 10th Edition diagnosis of I48.0, I48.1, I48.2, I48.3, I48.4, I48.91, I48.92, or ICD 9th Edition diagnosis of 427.31, 427.32, or a mention within an ECG report of “atrial fibrillation”, “atrial flutter”, “Afib”, “AFl”, “A-fib” or “A-fl” using text matching. While cases and controls were initially drawn from the ≥ 18-year-old population, prior to model development we restricted them to ≥ 40 years old to better target an at-risk population.

### Identification of controls

In each cohort, a simple random sample of patients without previously-identified AF the same size as the case cohort was identified. After restricting to ≥ 40-years-old, more cases than controls remained due to the generally older population of the cases. Controls were not matched to cases on specific variables to prevent bias and explore all possible relationships.

### Subjects

Patients were included in the development cohort if they were age ≥ 40 years on 1/1/2016 and had at least two clinical visits during the baseline period. The requirement for two clinical visits allowed for sufficient data for development and validation. Patients were excluded if they had an AF diagnosis prior to 1/1/2016 or had an unknown status of AF in the outcome period. The validation cohort was similarly defined with all criteria shifted 2 years earlier.

### Candidate Variable selection

Variables were selected as potential model predictors based on review of previous models as well as clinical expert opinion (authors TDI and NLC) on potential clinically-relevant variables. The complete list of variables initially considered for modeling is in Additional file 1: Table S1, including demographic characteristics, vital signs, social history, medical history, diagnoses, lab values, echocardiogram results, and imaging reports, including coronary angiography. Where appropriate and clinically meaningful, multiple variables were combined to create a “derived” variable to allow for overlapping of content, as shown in Additional file 1: Table S2. Appropriate transformations and coding based on clinical norms (Additional file 1: Table S3) were applied to the raw data; the recoded variables were used as candidates in model development. Missing data were explicitly categorized as such.

### Statistical methods for model development and validation

The primary analysis was conducted on the development data set based on candidate variables previously reported in the literature, resulting in a least absolute shrinkage and selection operator (LASSO)-penalized stepwise logistic regression model. The stepwise approach is described in Supplementary Methods, Additional file 1. The penalization methods, due to their shrinkage estimages, are robust to collinearity. This 10-variable model, labeled UNAFIED (Undiagnosed Atrial Fibrillation prediction using Electronic health Data), was run on the validation data set. We tested the sensitivity and robustness of the model extensively using two-way interactions and alternative data recoding schemes and against alternative models, including a more parsimonious 5-variable model and a “free-for-all” model that allowed the inclusion of previously unreported variables (see details in Supplementary Methods, Additional file 1). We also replicated two previous models (Volgman [12] and Aronson [11]) that used similar variables in large population-based cohorts on our data sets. The performance of all five models was compared using the C-statistic (area under the receiver operating characteristic curve, a measure of discrimination between cases and controls).

Risk scores were calculated for each patient based on the estimated parameters in the model results (Table 2). Youden’s Index was used to choose a threshold risk score, which optimized sensitivity and specificity for classifying patients into “higher” versus “lower” risk of having undiagnosed AF. All analyses were performed in SAS version 9.4 (High Performance).

### Proof-of-concept (POC) implementation

In partnership with our clinical affiliate, Eskenazi Health, we piloted implementation of the UNAFIED model as a non-interventional proof-of-concept, using pre-existing clinical decision support (CDS) tools within the Epic EHR system. Because of EHR limitations in using natural language parsing, we limited implementation to using structured EHR data only (i.e., no unstructured text searches) for POC. A sensitivity analysis was completed for excluding unstructured data from the original model work. Fewer than 1% of patients had term mentions in the simple NLP, and there was no appreciable change in C-statistic, which we believed acceptable for the pilot implementation. The CDS build was done by the Eskenazi Health Information Systems team and implemented for a 6-week trial run in late 2019. A rule-based AF risk score was added to an existing automated EHR registry that updated periodically for all patients in the health system. For patients aged ≥ 40 years without a diagnosis of AF who exceeded the risk threshold, a silent CDS alert was triggered at clinical encounters in the emergency department, hospital, and outpatient settings within Eskenazi Health. These silent CDS alerts were not displayed to clinicians during the pilot phase but instead enabled reporting on alerting patterns. Aggregate counts of patients identified by the alerts, along with selected clinical characteristics, were compared to patients with encounters but not identified as higher risk. In addition, we calculated the patient’s CHA_2_DS_2_-VASc [15] score, a prediction tool for assessing stroke risk in patients with non-rheumatic AF and used for guideline-recommended anti-coagulant use.

## Results

**Fig. 1:**
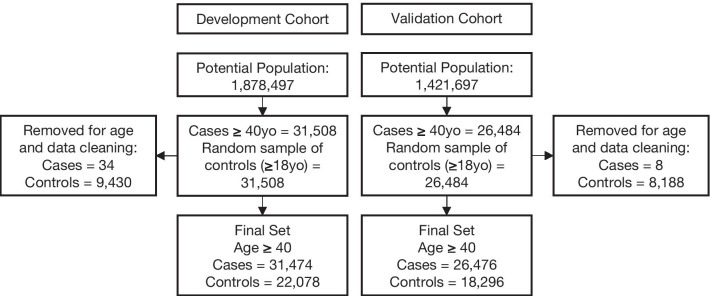
Model development cohorts

During the model development period, 31,474 patients within the retrospective cohort had a first AF diagnosis recorded within the two subsequent years of data (cases) while 1,295,281 patients did not (potential controls). Thus, the 2-year incidence of AF in our underlying population was 2.37%. A random sample of 22,078 controls was used in the model development. The validation cohort had 26,476 cases, and 18,296 controls randomly selected (Fig. 1). Table 1 shows patient characteristics for the model development cohort (see Additional file 1: Tables S7–S9, for model validation cohort characteristics). Average age for the AF cohort was 71.5 years with 49% women compared to 59.5 years for the non-AF cohort with 61% women. The comparisons of clinical variables retained in the final model are also presented in Table 1. (Further comparisons are in Additional file 1: Tables S3 and S4.)

**Table 1.** Characteristics of patients in development set

Variable	Overall N = 53552	AF (cases) N = 31474	No AF (controls) N = 22078	*P* value
Demographics				
Age, mean (SD), years	66.56 (13.42)	71.53 (11.87)	59.48 (12.28)	< .0001
Age, no. (%) years				< .0001
40–55	12474 (23.3%)	3287 (10.4%)	9187 (41.6%)	
56–66	13676 (25.5%)	6949 (22.1%)	6727 (30.5%)	
67–77	14822 (27.7%)	10630 (33.8%)	4192 (19.0%)	
> 77	12580 (23.5%)	10608 (33.7%)	1972 (8.9%)	
Sex				< .0001
Female	29044 (54.2%)	15561 (49.4%)	13483 (61.1%)	
Male	24507 (45.8%)	15913 (50.6%)	8594 (38.9%)	
Race, no. (%)				< .0001
White	41493 (77.5%)	24636 (78.3%)	16857 (76.4%)	
Black	3225 (6.0%)	1690 (5.4%)	1535 (7.0%)	
Other	8834 (16.5%)	5148 (16.4%)	3686 (16.7%)	
Ethnicity, no. (%)				< .0001
Not Hispanic or Latino	37219 (69.5%)	22681 (72.1%)	14538 (65.8%)	
Hispanic or Latino	828 (1.5%)	402 (1.3%)	426 (1.9%)	
Unknown	15505 (29.0%)	8391 (26.7%)	7114 (32.2%)	
Insurance type, No. (%)				< .0001
Commercial	28416 (53.1%)	16115 (51.2%)	12301 (55.7%)	
Medicaid	7324 (13.7%)	4289 (13.6%)	3035 (13.7%)	
Medicare	11141 (20.8%)	8432 (26.8%)	2709 (12.3%)	
Other/Unknown	6671 (12.5%)	2638 (8.4%)	4033 (18.3%)	
Additional diagnoses and laboratory variables included in final model				
Acute heart disease^a^, no. (%)	9782 (18.3%)	7874 (25.0%)	1908 (8.6%)	< .0001
Albumin < 3.5, no. (%), g/dL	4110 (7.7%)	3264 (10.4%)	846 (3.8%)	< .0001
Body mass index, no. (%), kg/m^2^				< .0001
Missing	33926 (63.4%)	18468 (58.7%)	15458 (70.0%)	
Normal weight: 18.5–24.9	4008 (7.5%)	2684 (8.5%)	1324 (6.0%)	
Obese: ≥ 30	9258 (17.3%)	6139 (19.5%)	3119 (14.1%)	
Overweight: 25–29.9	6013 (11.2%)	3926 (12.5%)	2087 (9.5%)	
Underweight: < 18.5	347 (0.6%)	257 (0.8%)	90 (0.4%)	
COPD, no. (%)	7891 (14.7%)	5909 (18.8%)	1982 (9.0%)	< . 0001
Kidney disease^b^, no. (%)	4783 (8.9%)	4060 (12.9%)	723 (3.3%)	< .0001
Shock, no. (%)	3365 (6.3%)	2593 (8.2%)	772 (3.5%)	

^a^If troponin > 0.04 or diagnosis of myocardial infarction

^b^BUN > 20 or (creatinine > 1.1 for female) or (creatinine > 1.3 for male) or (diagnoses of Chronic Kidney Disease or End Stage Renal Disease)

**Fig. 2:**
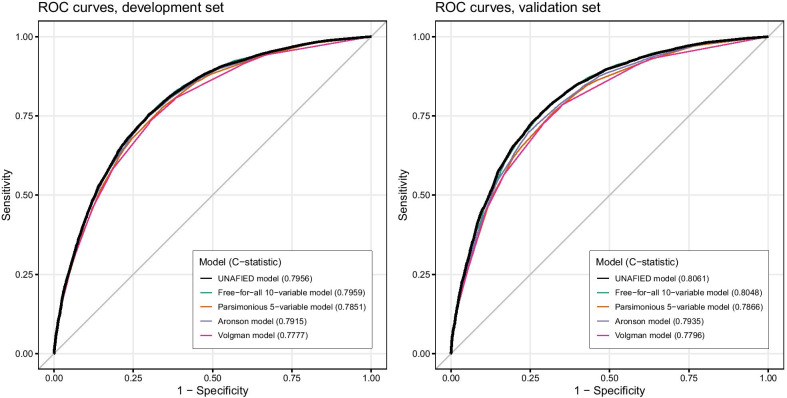
ROC curves for development and validation sets for three selected models in this study and comparison to two previous models

The final UNAFIED Model is displayed with its 10 variables and their parameter estimates (weights) in Table 2. The C-statistic of the development-phase UNAFIED Model (0.7956, 95% CI 0.7917–0.7994) could not be appreciably increased by any additional (including previously unreported) variables to enter the free-for-all model (C-statistic 0.7959, 95% CI 0.7921–0.7997), and the parsimonious 5-variable model (C-statistic 0.7851, 95% CI 0.7812–0.789) resulted in a more than one percentage point decrease. The C-statistics of two previous models (Volgman and Aronson) applied to our development data set were 0.7777 (95% CI 0.7738–0.7817) and 0.7915 (95% CI 0.7877–0.7954), respectively. The ROC curves of these five models are shown in Fig. 2. Comparison of all five models using the validation data set gave similar results (Fig. 2 and Additional file 1: Table S5). In the validation phase, our model achieved a C-statistic of 0.8061 (95% CI 0.802–0.8102).

**Table 2.** Logistic regression variable parameters

Parameter^a,b^	Description	Estimate^c^	Odds ratio (95% confidence interval)
Intercept		0.4063	
Age (years)	40 ≤ Age < 56	− 0.9741	0.38 (0.36, 0.4)
	67 ≤ Age < 77	0.7216	2.06 (1.94, 2.19)
	Age ≥ 77	1.5844	4.88 (4.54, 5.23)
Heart disease (derived)	Present	0.5053	1.66 (1.55, 1.77)
Albumin (g/dL)	Albumin < 3.5	0.7438	2.1 (1.93, 2.3)
BMI (kg/m^2^)	Missing	0.0884	1.09 (0.99, 1.21)
	BMI < 18.5	0.6086	1.84 (1.26, 2.69)
	24.9 ≤ BMI ≤ 29.9	0.0384	1.04 (0.92, 1.17)
	BMI > 29.9	0.3723	1.45 (1.3, 1.62)
COPD diagnosis	Present	0.5259	1.69 (1.57, 1.82)
Gender	Female	− 0.6226	0.54 (0.51, 0.56)
Heart failure diagnosis	Present	1.0609	2.89 (2.53, 3.31)
Insurance	Commercial	− 0.4111	0.66 (0.62, 0.71)
	Medicaid	0.0378	1.04 (0.95, 1.13)
	Other/unknown	− 0.8584	0.42 (0.39, 0.46)
Kidney disease (derived)	Present	0.58	1.79 (1.59, 2.01)
Shock diagnosis	Present	0.6219	1.86 (1.67, 2.08)

^a^See Additional file 1, for codes and logic used for extraction and derivation of parameters

^b^Reference parameters: Age 56–66 years (inclusive), no calculated heart disease, albumin ≥ 3.5 (or missing value), normal BMI (19.5–24.9), no COPD, male, no heart failure, Medicare insurance, no calculated kidney disease, and no shock.

^c^Each eligible patient had a risk score of exp(raw score)/(1 + exp(raw score)), where the raw score was the sum of the intercept and parameter estimates corresponding to the patients characteristics in each parameter.

Youden’s Index indicated an optimal threshold score of 0.591, which achieved 74% sensitivity and 74% specificity in the development cohort. Combining this with the underlying population’s 2-year AF diagnosis incidence (2.37%), we estimated identifying 27.1% of the patients in the underlying population as “higher risk.”

During the 6-week silent POC there were 22,272 unique patients ≥ 40 years old identified through encounters (e.g., emergency, inpatient, or outpatient visit). Using the threshold described above (rounded to 0.6 for implementation), 7916 (35.5%) of these patients were identified as higher-risk in at least one encounter. These patients were identified in the emergency department (1705), inpatient (1793), and outpatient (7723) settings (one patient may be identified in multiple locations). Nearly half of patients identified in an outpatient setting were in the primary care clinics. Patients with “higher-risk” flags were labeled “UNAFIED” patients. In Table 3, we present the demographic characteristics of all patients in the implementation pilot, including UNAFIED and non-UNAFIED patients (who had a visit but did not trigger any flags).

**Table 3.** Characteristics of patients in the proof-of-concept implementation

	UNAFIED patients	Non-UNAFIED patients	All
No.	7916	14356	22272
Race, no. (%)			
Black or African American	4129 (52.16%)	6065 (42.25%)	10194 (45.77%)
White	3009 (38.01%)	5189 (36.15%)	8198 (36.81%)
Unknown	451 (5.70%)	2114 (14.73%)	2565 (11.52%)
More than one race	155 (1.96%)	530 (3.69%)	685 (3.08%)
Native Hawaiian or other Pacific Islander	45 (0.57%)	202 (1.41%)	247 (1.11%)
Asian	109 (1.38%)	217 (1.51%)	326 (1.46%)
American Indian or Alaska Native	18 (0.23%)	39 (0.27%)	57 (0.26%)
Ethnicity, no. (%)			
Not Hispanic, Latino/a, or Spanish origin	7171 (90.59%)	10913 (76.02%)	18084 (81.20%)
Hispanic or Latino	595 (7.52%)	2982 (20.77%)	3577 (16.06%)
Unknown	150 (1.89%)	461 (3.21%)	611 (2.74%)
Age, no. (%), years			
40–44	117 (1.48%)	3222 (22.44%)	3339 (14.99%)
45–54	445 (5.62%)	5968 (41.57%)	6413 (28.79%)
55–64	2981 (37.66%)	4203 (29.28%)	7184 (32.26%)
65–74	2815 (35.56%)	750 (5.22%)	3565 (16.01%)
75–84	1175 (14.84%)	152 (1.06%)	1327 (5.96%)
85+	383 (4.84%)	61 (0.42%)	444 (1.99%)
Age, mean (SD), years	66.46 (9.82)	51.99 (8.53)	57.14 (11.37)
Sex, no. (%)			
Female	3930 (49.65%)	9113 (63.48%)	13043 (58.56%)
Male	3986 (50.35%)	5243 (36.52%)	9229 (41.44%)
CHA_2_DS_2_-VASc, mean (SD)	2.557 (1.83)	1.213 (1.40)	1.691 (1.69)

UNAFIED patients had, on average, a higher CHA_2_DS_2_-VASc score (mean 2.5, SD 1.8) than non-UNAFIED patients (mean 1.2, SD 1.4, Table 3). They were generally older, non-Hispanic, and evenly split between male and female. Additional file 1: Table S6, compares the proportion of patients with CHA_2_DS_2_-VASc score ≥ 2 between UNAFIED and non-UNAFIED patients, overall and within age and sex strata. Generally, UNAFIED patients had higher proportions of patients with CHA_2_DS_2_-VASc score ≥ 2 in almost all strata.

## Discussion

Our study was able to successfully develop and validate a 2-year undiagnosed AF risk model with predictive capability at least as good as those previously reported, using commonly available EHR data, and build a proof of concept implementation within an existing common EHR system. Previous models have been developed either in limited community-based cohorts, used longer prediction horizons, or included variables not routinely available as structured data fields within an EHR. Additionally, they were not tested for practical implementation. The UNAFIED model used data elements available as structured data fields within the EHR, commonly collected in multiple practice settings, yet still accommodates reasonably incomplete data sources.

Our model shares several risk predictors with previous models including age, sex, acute heart disease, COPD, heart failure, and BMI. In contrast to previous models that only associated overweight with AF risk, we additionally found low BMI (< 18.5) in this population was a predictor of AF diagnosis in the subsequent 2-year time period. Chronic kidney disease, low albumin, hypovolemic shock, and insurance type were predictors of AF risk unique to this model. Patients with Medicaid insurance had higher risk when compared to patients with commercial insurance or Medicare. This may be related to underserved or low socioeconomic status persons having less access to care and diagnostic services. However, insurance practice and enrollment patterns can vary based on geographic location, so the generalizability and clinical significance of this variable are yet to be determined.

Our implementation identified 36% of patients screened during the implementation phase, compared to an expected 27% based on model development. We suspect this is due to age, insurance, and other differences in the implementation population (safety-net urban hospital) and development population (regional HIE). Additionally, our implementation allowed multiple opportunities for the model to identify a patient, whereas the development and validation phases used a single time point. Mean CHA_2_DS_2_-VASc score for UNAFIED patints was higher than non-UNAFIED patients suggesting the ability of the model to identify patients with opportunities for guideline-recommended management to potentially prevent devastating health outcomes.

The choice of a threshold predicted risk score is an important step in implementing this model and depends on several factors. For our implementation, we optimized model sensitivity and specificity. Raising the cutoff reduces sensitivity but increases specificity, and vice versa. Considering finite resources, a higher threshold will identify a smaller group of “higher risk” patients. The estimated prevalence of undiagnosed AF being predicted is another factor. Finally, an automated screening mechanism that uses human-facing decision support should weigh the volume of alerts (including inevitable false positives) and their effect on clinician well-being and performance versus other ways for clinicians to receive the information in their workflows. Broadly-speaking, institutions considering implementing any predictive model generally will want to consider these various factors [16]. A note of caution: the model performance described here is based on the population used to develop it and the chosen threshold score. Additional health systems should verify performance in their own setting.

Screening recommendations for asymptomatic and undiagnosed adults for AF range from no recommendation (USPSTF, insufficient evidence for using ECG, compared to pulse palpation [17]) to opportunistic screening (European Society of Cardiology [18]) to systematic screening (American Heart Association and American Stroke Association [3]). We note organizational recommendations have variations too nuanced to summarize here. Additionally, recent results show a dramatically increasing number needed to screen based on age for identifying undiagnosed AF [19]. Our study demonstrates the feasibility of identifying patients at higher risk of AF who are potential candidates for further screening or evaluation to detect and diagnose previously-undiagnosed AF. Ultimately, our primary goal is for accurate diagnosis and guideline-recommended management, potentially avoiding stroke or systemic embolization. Targeted screening may enhance the diagnostic yield through a personalized, stepwise approach. For example, a predictive algorithm using only EHR data can serve as a precursor (via targeted screening) or a companion to processes shown in recent work with wearable devices for identifying AF [20, 21]. However, in further support of EHR-data algorithms, relying on wearables alone requires the burden of cost on the healthcare system, or relies on a socially inequitable approach of relying on consumer purchasing.

To our knowledge, the ability to identify patients prior to the clinical diagnosis of AF has not been scaled within a healthcare system. Our model allows for multiple avenues of interventions including clinician alerts, patient portal notifications, or a referral to a population health coordinator. Separately, we found patients identified by the encounter-based approach to be similar to patients incidentally identified without an encounter, which supports efforts for a population health approach without an in-person visit. An intervention may preempt the sentinel stroke that is associated with AF for the first time in approximately 20% of patients [22]. Given a fivefold increased risk of stroke in patients with AF compared to those without AF [1]] and the preventive potential of guideline-recommended treatments, identifying these patients with a risk of undiagnosed AF using EHR data at both patient and population levels may result in a significant benefit to them and their families.

There are limitations of our study. The first is that the model was developed and validated with clinical data in an Indiana-based HIE, and may not be applicable to all health systems’ demographics. We have attempted to account for this by comparing our model to previous models for AF identification. Additionally, the model may not be applicable to countries with a different health coverage or insurance environment; further investigation is needed to address this parameter in future work. A second limitation is in our ability to identify patients with limited to no previous clinical data. Another limitation is in our ability to recommend a follow-up assessment(s) or intervention(s) for when a patient is identified as higher-risk for undiagnosed AF. In our study we elected to do a proof-of-concept implementation with a silent alert and not pursue a specific method for further monitoring, screening, or intervention for confirmatory testing of AF.

We recommend this model be tested and implemented within additional healthcare systems, with individual or algorithmic decisions on how to interpret the risk score for reasonable follow-up or clinical intervention. This may help calculate the patient- and system-level value of identifying undiagnosed AF patients to reduce their risk of stroke or systemic embolism. Future studies may also use sophisticated natural language processing to evaluate unstructured data to explore an expanded risk prediction.

## Conclusion

Utilizing only existing electronic health data, this parsimonious model for identifying risk of undiagnosed AF over 2 years achieved a C-statistic of 0.81. It is among the highest reported in literature to date seen, and was successfully implemented into a production EHR in a large metropolitan health system. Implementing this model in additional health systems could facilitate identification of patients at higher-risk of undiagnosed AF, leading to further assessment to evaluate for AF. Guideline-recommended interventions may help reduce these patients’ risk of stroke or systemic embolism. This non-invasive, inexpensive screening approach may benefit patients and their families, and the health systems caring for them.

## Supplementary Information

**Additional file 1**. Supplementary tables and methods describing UNAFIED model development and results.

## Data Availability

The data that support the findings of this study are available from Regenstrief Institute, Inc., but restrictions apply to the availability of these data, which were used under agreement for the current study, and so are not publicly available. However, data are available upon reasonable request to Regenstrief Data Services, Regenstrief Institute, Inc, 1101 W Tenth St, Indianapolis, IN, 46202 (https://www.regenstrief.org/rds/services/). Detailed methods have been included in the manuscript and Additional file 1.

## Abbreviations

AF: Atrial fibrillation
AFl: Atrial flutter
ARIC: Atherosclerosis Risk in Communities
BMI: Body mass index
CDS: Clinical decision support
CHARGE-AF: Cohorts for Heart and Aging Research in Genomic Epidemiology Atrial Fibrillation Consortium
COPD: Chronic Obstructive Pulmonary Disease
ECG: Electrocardiogram
EHR: Electronic health record
FHS: Framingham heart study
HIE: Health information exchange
ICD: International Statistical Classification of Diseases and Related Health Problems
INPC: Indiana Network for Patient Care
LASSO: Least absolute shrinkage and selection operator
NLP: Natural language processing
POC: Proof-of-concept
ROC: Receiver-operating characteristics
STROBE: Strengthening the Reporting of Observational Studies in Epidemiology
TRIPOD: Transparent Reporting of a Multivariable Prediction Model for Individual Prognosis or Diagnosis
UNAFIED: Undiagnosed Atrial Fibrillation prediction using Electronic health Data
USPSTF: United States Preventive Services Task Force
