# Supplementary material for: Development, validation, and proof-of-concept implementation of a two-year risk prediction model for undiagnosed atrial fibrillation using common electronic health data (UNAFIED)
Source: BMC Med Inform Decis Mak. 2021 Apr 3;21:112. doi: 10.1186/s12911-021-01482-1 (PMC8019173; doi:10.1186/s12911-021-01482-1)
Supplement: Supplementary file 1 — Additional file 1. Supplementary tables and methods describing UNAFIED model development and results. [file 12911_2021_1482_MOESM1_ESM.docx]

**Additional File 1 for:**

Development and proof-of-concept implementation of the Undiagnosed Atrial Fibrillation prediction using Electronic health Data (UNAFIED) model

Randall W. Grout, MD, MS^1,2^

Siu L. Hui, PhD^1,3,4^

Timothy D. Imler, MD^1,5^

Sarah El-Azab, MS^3^

Jarod Baker, MBA^1^

George H. Sands, MD^6^

Mohammad Ateya, PharmD, MS^6^

Francis Pike, PhD^4^

1. Center for Biomedical Informatics; Regenstrief Institute. Indianapolis, IN
2. Department of Pediatrics; Indiana University School of Medicine. Indianapolis, IN
3. Research Services; Regenstrief Institute. Indianapolis, IN
4. Department of Biostatistics; Indiana University School of Medicine. Indianapolis, IN
5. Division of Gastroenterology and Hepatology; Indiana University School of Medicine. Indianapolis, IN
6. Pfizer Inc, US Medical Affairs New York, NY

**Corresponding Author:**

Randall Grout, MD, MS

Email: rgrout@iu.edu

1101 W Tenth St

Indianapolis, IN 46202

**Contents**

Table S1. Variables utilized in model development.

Table S2. Derived variables utilized in model development.

Table S3. Laboratory values in model development data set.

Table S4. Comorbidities in model development data set.

Table S5. Model performance in development and validation sets

Table S6. CHA₂DS₂-VASc descriptive statistics among UNAFIED and non-UNAFIED patients

Table S7. Laboratory values in model validation data set.

Table S8: Comorbidities in model validation data set.

Table S9: Demographics in the model validation set.

Supplementary Methods.

TRIPOD Statement.

**Table S1. Variables utilized in model development.**

***Demographics***

| Variable | Variable Type | Variable Details | Notes and Logic | Coding |
| --- | --- | --- | --- | --- |
| Age | Numeric | Patient age. | Patient age at start of outcome period (i.e. as of 1/1/2016 for development cohort and 1/1/2014 for validation cohort). Extracted with local codes/concepts from HIE data | 40-55 =0  56-66=1  67-76=2  >76=3 |
| Sex | Character | F=Female, M=Male, U=Unknown | Extracted with local codes/concepts from HIE data |  |
| Race | Character | White, Hispanic/Latino, Other, Unknown, Asian, Native Hawaiian, or Pacific Islander, Black or African American, American Indian or Alaska Native, Multiracial | To correlate potential ethnographic determinants of AF/AFl risk and/or diagnosis. Extracted with local codes/concepts from HIE data. | White= ‘White’  Black or African American=’Black’  All other categories Coded as ‘Other’ |
| Ethnicity | Character | Hispanic or Latino, Not Hispanic or Latino, Unknown | See race above. Extracted with local codes/concepts from HIE data |  |
| Urbanicity | Numeric | Urbanicity score from 0-9, with 0 being very rural and 9 being very urban. | Zip code extracted with local codes/concepts from HIE data and mapped to McKinsey Urbanicity Index. To determine whether site of care / residence affects AF diagnosis / risk. |  |
| Insurance | Character | Commercial, Medicaid, Medicare, Self Pay, Sliding Fee, Workers Comp, Other/Unknown | Insurance used at encounter for diagnosis of AF (or closest encounter to date of term mention). Extracted with local codes/concepts from HIE data | Reclassed as:  Commercial=’Commercial’  Medicaid=’Medicaid’  Medicare=’Medicare’  Missing or other =’Other/Unknown’ |

***Vitals***

| Variable | Variable Type | Variable Details | Notes and Logic | Coding |
| --- | --- | --- | --- | --- |
| Height | Numeric | Structured test results | Extracted with local codes/concepts from HIE data | Not used due to low yield |
| Weight | Numeric | Structured test results | Extracted with local codes/concepts from HIE data | Not used due to low yield |
| BMI | Numeric | Structured test results | Calculated from height and weight. Calculation prioritized and, if height/weight data not available, BMI field entry used next. | Missing=’-1’ ‘Normal’  <18.5=’0’ ‘Underweight’  18.5-24.9=’1’ ‘Normal’  24.9 -29.9=’2’ Overweight’  >30 =’3’ ‘Obese’ |
| Systolic Blood Pressure | Numeric | Structured test results | Extracted with local codes/concepts from HIE data | Dropped due to Low yield |
| Diastolic Blood Pressure | Numeric | Structured test results | Extracted with local codes/concepts from HIE data | Dropped due to Low yield |

***Social History***

| Variable | Variable Type | Variable Details | Notes and Logic | Coding |
| --- | --- | --- | --- | --- |
| Cocaine Use | Numeric | 1=Yes, 0=No | Amphetamine use is proarrhythmic and may confer increased AF risk. Most available illicit use is cocaine. Flagged as the presence of the following ICD codes and term mentions:   - Cocaine related disorders (ICD9: 304.2, 305.6 , ICD10: F14) - Term mentions in clinical notes: “cocaine” | Dropped due to low yield |
| Alcohol Use | Numeric | 1=Yes, 0=No | Alcohol is also proarrhythmic. Flagged as the presence of the following ICD codes and term mentions:   - Alcohol related disorders (ICD9: 305.0, 303, ICD10: F10) - Term mentions in clinical notes: “Alcohol”, “EtOH”, “Ethanol”, “Drunk”, “CAGE (questionnaire)” |  |
| Tobacco Use | Numeric | 1=Yes, 0=No | Tested and included in UK/CHARGE-F/ARIC/Framingham risk scores. Flagged as the presence of the following ICD codes and term mentions:   - History of tobacco use (ICD9: V69.8, V15.82 ICD10: Z72.0, Z87.891) - Nicotine dependence (ICD9: 305.1, ICD10: F17) - Term mentions in clinical notes: “smoking”, “smoker” NOT “nonsmoking” |  |

***Medical History***

| Variable | Variable Type | Variable Details | Notes and Logic | Coding |
| --- | --- | --- | --- | --- |
| Vaccinations | Numeric | 1=Yes, 0=No | All CPT and NDC codes mapped from CDC CVX codes. To correlate with presence / absence of vaccination. |  |
| Chemo Drugs | Numeric | 1=Yes, 0=No | Cardiomyopathic chemotherapy may confer risk. Term mentions in clinical notes: “doxorubicin”, “anthracycline”, “ibrutinib” | Dropped due to Low Yield |
| Hypertension medication | Numeric | 1=Yes, 0=No | To correlate hypertension with AF risk. Included in ARIC and Framingham risk scores. Medication fills defined via Generic Product Identifier (GPI) code:   - 36 (except 3610, 3615, 362020), 3210, 3330, 3720, 3740, 3750, 3760 |  |
| 2 ED Visits | Numeric | 1=Yes, 0=No | Record of 2 emergency visits/admission with 2 years of index date. ED presentations may hide / present occult AF incidence. |  |
| Holiday Heart | Numeric | 1=Yes, 0=No | Record of ED visit within 1 week of July 4^th^, Thanksgiving, or Christmas. Commonly associated with AF risk. |  |

***Medical History: Diagnoses***

| Variable | Variable Type | Variable Details | Notes and Logic | Coding |
| --- | --- | --- | --- | --- |
| Inflammatory/immune disease | Numeric | 1=Yes, 0=No | Flagged as the presence of the following ICD codes and term mentions:   - Lupus (ICD9: 694.5, ICD10: L93.X) - Ankylosing spondylitis (ICD9: 720.X, ICD10: M45.X) - Rheumatoid arthritis (ICD9: 714.X, ICD10: M05.X) - Crohn’s (ICD9: 555.X, ICD10: K50.X) - Ulceritive colitis (ICD9: 556.X, ICD10: K51.X) - Inflammatory bowel disease (captured through ICD codes for Crohn’s and ulcerative colitis) - Sarcoidosis (ICD9: 135, ICD10: D86.X) - Term mentions in clinical notes: “lupus”, “ankylosing spondylitis”, “rheumatoid arthritis”, “crohn’s”, “lzheimer colitis”, “inflammatory bowel disease”, “sarcoidosis” |  |
| Precordial murmur | Numeric | 1=Yes, 0=No | May signify valvular disease that confers cardiomyopathy +/- arrhythmia risk. Flagged as the presence of the following ICD codes and term mentions:   - Other cardiac sounds (ICD9: 785.3, ICD10: R01.2) - Term mentions in clinical notes: “precordial murmur” | Dropped due to low Yield |
| Chronic ischemic heart disease | Numeric | 1=Yes, 0=No | Ischemia may confer cardiomyopathy and/or be proarrhythmic itself. Flagged as the presence of the following ICD codes and term mentions:   - ICD9: 414.X*, ICD10: I25.X* - Term mentions in clinical notes: “chronic ischemic heart disease” |  |
| Hypertension | Numeric | 1=Yes, 0=No | Hypertension known clinical AF risk factor. Flagged as the presence of the following ICD codes and term mentions:   - Hypertensive diseases (ICD9: 401.X*, 402.X, 403.X, 404.X, 405.X ICD10: I10.X, I11.X, I12.X, I13.X, I15.X, I16.X*) - Term mentions in clinical notes: “hypertension”, “hypertensive” |  |
| Shock | Numeric | 1=Yes, 0=No | Flagged as the presence of the following ICD codes and term mentions:   - Shock, not elsewhere classified (ICD9: 785.X, ICD10: R57.X) - Term mentions in clinical notes: “shock”, “hypovolemic”, “hypotension” |  |
| Chronic kidney disease | Numeric | 1=Yes, 0=No | Flagged as the presence of the following ICD codes and term mentions:   - ICD9: 585.X*, ICD10: N18.X* - Term mentions in clinical notes: “chronic kidney disease”, “CKD” |  |
| End-stage renal disease | Numeric | 1=Yes, 0=No | Flagged as the presence of the following ICD codes and term mentions:   - ICD9: 585.6*, ICD10: N18.6* - Term mentions in clinical notes: “end-stage renal disease”, “ESRD”, “dialysis”, “hemodialysis” |  |
| Diabetes mellitus | Numeric | 1=Yes, 0=No | See “A1c” below. Flagged as the presence of the following ICD codes and term mentions:   - ICD9: 250.X*, ICD10: E08.X, E09.X, E10.X, E11.X*, E13.X* - Term mentions in clinical notes: “diabetes mellitus”, “diabetes”, NOT “diabetes insipidus” |  |
| Osteoarthritis | Numeric | 1=Yes, 0=No | Flagged as the presence of the following ICD codes and term mentions:   - ICD9: 715.X, ICD10: M15.X, M16.X, M17.X, M18.X, M19.X - Term mentions in clinical notes: “osteoarthritis” |  |
| Chronic respiratory failure | Numeric | 1=Yes, 0=No | Flagged as the presence of the following ICD codes and term mentions:   - ICD9: 518.X, ICD10: J96.X - Term mentions in clinical notes: “chronic respiratory failure” |  |
| Alzheimer’s disease | Numeric | 1=Yes, 0=No | Flagged as the presence of the following ICD codes and term mentions:   - ICD9: 331, ICD10: G30 - Term mentions in clinical notes: “lzheimer’s” | Dropped due to low Yield |
| Chronic obstructive pulmonary disease | Numeric | 1=Yes, 0=No | Flagged as the presence of the following ICD codes and term mentions:   - COPD (ICD9: 327.23*, 491.X*, 492.X, 493.X, 496* ICD10: G47.33, J41.X, J42, J43.X, J44.X*) - Term mentions in clinical notes: “COPD” / “chronic obstructive pulmonary disease” |  |
| Peripheral vascular disease | Numeric | 1=Yes, 0=No | Flagged as the presence of the following ICD codes and term mentions:   - Diseases of arteries, arterioles, capillaries (ICD9: 440.X, 441.X, 442.X, 443.X*, 444.X, 445.X, 446.X, 447.X, 448.X, 449, ICD10: I70.X, I71.X, I72.X, I73.X*, I74.X, I75.X, I76, I77.X, I78.X, I79.X) - Term mentions in clinical notes: “PVD”, “peripheral vascular disease”, “peripheral arterial disease”, “PAD” |  |
| Paralysis | Numeric | 1=Yes, 0=No | Flagged as the presence of the following ICD codes and term mentions:   - Other paralytic symptoms (ICD9: 344.X, ICD10: G83.X) - Term mentions in clinical notes: “paralysis” | Dropped due to low yield |
| Sleep apnea | Numeric | 1=Yes, 0=No | Flagged as the presence of the following ICD codes and term mentions:   - ICD9: 327.20, 327.23*,780.51, 780.53, 780.57, ICD10: G47.33*, G47.30 - Term mentions in clinical notes: “sleep apnea” |  |
| Sickle cell disease | Numeric | 1=Yes, 0=No | Flagged as the presence of the following ICD codes and term mentions:   - ICD9: 282.4X, 282.5, 282.6X, ICD10: D57.X - Term mentions in clinical notes: “sickle cell” | Drooped due to Low Yield |
| Heart attack/heart disease | Numeric | 1=Yes, 0=No | Flagged as the presence of the following ICD codes and term mentions:   - Ischemic heart diseases (ICD9: 410.X*, 411.X, 412, 413.X*, 414.X*, 429.71*, 429.79*, 996.03*, V45.0X*, V45.81*, V45.82* ICD10: I20.X*, I21.X*, I22.X*, I23.X*, I24.X*, I25.X*, I51.0*, T82.X*, Z95.X*, Z98.61*) - Term mentions in clinical notes: “Coronary artery disease”, “coronary heart disease”, “Myocardial infarction”, “MI”, “heart attack” |  |
| Heart failure | Numeric | 1=Yes, 0=No | Associated with AF.   - Tachycardia induced cardiomyopathy (ICD9: 425.X, ICD10: I42.X) - Heart failure (ID9: 428.X, ICD10: I50.X) - Term mentions in clinical notes: heart failure, tachycardia induced cardiomyopathy |  |
| Stroke | Numeric | 1=Yes, 0=No | - Stroke (ICD9: 433.X, 434.X, ICD10: I63.X) - Transient ischemic attack (ICD9: 433.10, ICD10: G45.8, G45.9) - Fainting (ICD9: 780.0X, ICD10: R55) - Dizziness (ICD9: 780.4, ICD10: R42) | Dropped due to Low Yield |

***Lab Values***

| Variable | Variable Type | Variable Details | Notes and Logic | Coding |
| --- | --- | --- | --- | --- |
| Sodium (Na) | Numeric | Structured test results | Free water balance may confer AF risk. Extracted with local codes/concepts from HIE data  The most recent value prior to index date | Missing=’1’ ‘Normal’  <136 =’0’ ‘Low’  136-145=’1’ ‘Normal’  >145=’3’ ‘High’ |
| Min Sodium | Class | Created | The minimum value within the baseline period | Missing=’1’ ‘Normal’  <136 =’0’ ‘Low’  136-145=’1’ ‘Normal’  >145=’3’ ‘High’ |
| Max Sodium | Class | Created | The maximum value within the baseline period | Missing=’1’ ‘Normal’  <136 =’0’ ‘Low’  136-145=’1’ ‘Normal’  >145=’3’ ‘High’ |
| Potassium (K) | Numeric | Structured test results | Proarrhythmic. Extracted with local codes/concepts from HIE data  The most recent value prior to index date | Missing=’1’ ‘Normal’  <3.5 =’0’ ‘Low’  3.5-5.0=’1’ ‘Normal’  >5.0 =3 ‘High’ |
| Min Potassium | Class | Created | The minimum value within the baseline period | Missing=’1’ ‘Normal’  <3.5 =’0’ ‘Low’  3.5-5.0=’1’ ‘Normal’  >5.0 =3 ‘High’ |
| Max Potassium | Class | Created | The maximum value within the baseline period | Missing=’1’ ‘Normal’  <3.5 =’0’ ‘Low’  3.5-5.0=’1’ ‘Normal’  >5.0 =3 ‘High’ |
| Blood urea nitrogen (BUN) | Numeric | Structured test results | BUN associated with renal failure (a known AF risk factor). Extracted with local codes/concepts from HIE data | Missing=’1’ ‘Normal’  <8 =’0’ ‘Low’  8-20=’1’ ‘Normal’  >20 = ‘3’ ‘High’ |
| Creatinine (Cr) | Numeric | Structured test results | Cr associated with renal failure (a known AF risk factor). Extracted with local codes/concepts from HIE data  The most recent value prior to index date | Missing=’1’ ‘Normal’  <0.5 and Female=’0’ ‘Low’  0.5-1.10 and Female=’1’ ‘Normal’  > 1.10 and Female=’3’ ‘High’    <0.7 and Male=’0’ ‘Low’  0.7-1.3 and Male=’1’ ‘Normal’  > 1.3 and Male=’3’ ‘High’ |
| Min Creatinine | Class | Created | The minimum value within the baseline period | Missing=’1’ ‘Normal’  <0.5 and Female=’0’ ‘Low’  0.5-1.10 and Female=’1’ ‘Normal’  > 1.10 and Female=’3’ ‘High’    <0.7 and Male=’0’ ‘Low’  0.7-1.3 and Male=’1’ ‘Normal’  > 1.3 and Male=’3’ ‘High’ |
| Max Creatinine | Class | Created | The maximum value within the baseline period | Missing=’1’ ‘Normal’  <0.5 and Female=’0’ ‘Low’  0.5-1.10 and Female=’1’ ‘Normal’  > 1.10 and Female=’3’ ‘High’    <0.7 and Male=’0’ ‘Low’  0.7-1.3 and Male=’1’ ‘Normal’  > 1.3 and Male=’3’ ‘High’ |
| Calcium (Ca) | Numeric | Structured test results | Proarrhythmic. Extracted with local codes/concepts from HIE data | Missing=’1’ ‘Normal’  <8.6 =’0’ ‘Low’  8.6-10.2 =’1’ ‘Normal’  >10.2 =’3’ ‘High’ |
| Magnesium (Mg) | Numeric | Structured test results | Proarrhythmic. Extracted with local codes/concepts from HIE data | Missing=’1’ ‘Normal’  <1.6=’0’ ‘Low’  1.6-2.6=’1’ ‘Normal’  >2.6 =’3’ ‘High’ |
| Hemoglobin (Hgb) | Numeric | Structured test results | Anemia associated with arrhythmia. Extracted with local codes/concepts from HIE data  The most recent value prior to index date | Missing=’1’ ‘Normal’  <12 and Female=’0’ Low’  12-16 and Female=’1’ ‘Normal’  >16 and Female=’3’ ‘High’    <14 and Male=’0’ Low’  14-18 and Male=’1’ ‘Normal’  >18 and Male=’3’ ‘High’ |
| Min Hemoglobin | Class | Created | The minimum value within the baseline period | Missing=’1’ ‘Normal’  <12 and Female=’0’ Low’  12-16 and Female=’1’ ‘Normal’  >16 and Female=’3’ ‘High’    <14 and Male=’0’ Low’  14-18 and Male=’1’ ‘Normal’  >18 and Male=’3’ ‘High’ |
| Max Hemoglobin | Class | Created | The maximum value within the baseline period | Missing=’1’ ‘Normal’  <12 and Female=’0’ Low’  12-16 and Female=’1’ ‘Normal’  >16 and Female=’3’ ‘High’    <14 and Male=’0’ Low’  14-18 and Male=’1’ ‘Normal’  >18 and Male=’3’ ‘High’ |
| Hematocrit | Numeric | Structured test results | Anemia associated with arrhythmia. Extracted with local codes/concepts from HIE data  The most recent value prior to index date | Missing=’1’ ‘Normal’  <37 and Female=’0’ ‘Low’  37-47 and Female=’1’ ‘Normal’  >47 and Female =’3’ ‘High’    <42 and Male=’0’ ‘Low’  42-50 and Male=’1’ ‘Normal’  >50 and Male =’3’ ‘High’ |
| Min Hematocrit | Class | Created | The minimum value within the baseline period | <37 and Female=’0’ ‘Low’  37-47 and Female=’1’ ‘Normal’  >47 and Female =’3’ ‘High’    <42 and Male=’0’ ‘Low’  42-50 and Male=’1’ ‘Normal’  >50 and Male =’3’ ‘High’ |
| Max Hematocrit | Class | Created | The maximum value within the baseline period | <37 and Female=’0’ ‘Low’  37-47 and Female=’1’ ‘Normal’  >47 and Female =’3’ ‘High’    <42 and Male=’0’ ‘Low’  42-50 and Male=’1’ ‘Normal’  >50 and Male =’3’ ‘High’ |
| Platelet count | Numeric | Structured test results | Associated with inflammation. Extracted with local codes/concepts from HIE data | Missing=’1’ ‘Normal’  <150 =’0’ ‘Low’  150-450=’1’ ‘Normal’  >450 =’3’ ‘High’ |
| Neutrophils | Numeric | Structured test results | Associated with inflammation. Extracted with local codes/concepts from HIE data | Missing=’1’ ‘Normal’  <50=’0’ ‘Low’  50-70=’1’ ‘Normal’  >70 =’3’ ‘High’ |
| Lymphocytes | Numeric | Structured test results | Associated with inflammation. Extracted with local codes/concepts from HIE data | Missing=’1’ ‘Normal’  <30 =’0’ ‘Low’  30-45=’1’ ‘Normal’  >45=’3’ ‘High’ |
| Cortisol | Numeric | Structured test results | Proarrhythmic (or at least associated with hypercatacholaminergia). Extracted with local codes/concepts from HIE data | Not included due to low yield |
| Plasma metanephrines | Numeric | Structured test results | Proarrhythmic (or at least associated with hypercatacholaminergia). Extracted with local codes/concepts from HIE data | Not included due to low yield |
| Parathyroid hormone (PTH) | Numeric | Structured test results | Associated with calcium homeostasis (potentially proarrhythmic). Extracted with local codes/concepts from HIE data | Missing=’1’ ‘Normal’  <10 =’0’ ‘Low’  10-65=’1’ ‘Normal’  >65 =’3’ ‘High’ |
| TSH | Numeric | Structured test results | Proarrhythmic when low (i.e., hyperthyroid). Extracted with local codes/concepts from HIE data | Missing=’1’ ‘Normal’  <0.5 =’0’ ‘Low’  0.5-4.0 =’1’ ‘Normal’  >4.0 =’3’ ‘High’ |
| Troponin | Numeric | Structured test results | Associated with cardiac damage /ischemia. Extracted with local codes/concepts from HIE data | Missing=’1’ ‘Normal’  <=0.04 =’1’ ‘Normal’  >0.04 =’3’ ‘High’ |
| Total bilirubin | Numeric | Structured test results | Associated with heart failure / overload. Extracted with local codes/concepts from HIE data | Missing=’1’ ‘Normal’  <0.3 =’0’ ‘Low’  0.3-1.0=’1’ ‘Normal’  <1=’3’ ‘High’ |
| Albumin | Numeric | Structured test results | Indicative of poor nutrition, that may be incident with other proarrhythmic factors. Extracted with local codes/concepts from HIE data | Missing=’1’ ‘Normal’  <3.5=’0’ ‘Low’  3.5-5.5=’1’ ‘Normal’  >5.5=’3’ ‘High’ |
| AST | Numeric | Structured test results | See “total bilirubin” above. Extracted with local codes/concepts from HIE data | Missing=’1’ ‘Normal’  <10 =’0’ ‘Low’  10-40=’1’ ‘Normal’  >40 =’3’ ‘High’ |
| ALT | Numeric | Structured test results | See “total bilirubin” above. Extracted with local codes/concepts from HIE data | Missing=’1’ ‘Normal’  <10 =’0’ ‘Low’  10-40=’1’ ‘Normal’  >40 =’3’ ‘High’ |
| Alkaline phosphatase | Numeric | Structured test results | See “total bilirubin” above. Extracted with local codes/concepts from HIE data | Missing=’1’ ‘Normal’  <30=’0’ ‘Low’  30-120=’1’ ‘Normal  >120 =’3’ ‘High’ |
| Hemoglobin A1C | Numeric | Structured test results | Associated with diabetes control (confers post-AF stroke risk). Extracted with local codes/concepts from HIE data | Missing=’1’ ‘Normal’  <=5.7 =’1’ ‘Normal’  >5.7- <=6.4 =’2’ ‘Borderline High’  >6.4 =’3’ ‘High’ |
| Total cholesterol | Numeric | Structured test results | May confer ischemia risk. Extracted with local codes/concepts from HIE data | Missing=’1’ ‘Normal’  <200 =’1’ ‘Normal’  >=200 =’3’ ‘High’ |
| HDL | Numeric | Structured test results | May confer ischemia risk. Extracted with local codes/concepts from HIE data | Missing=’1’ ‘Normal’  >=50 and Female=’1’ ‘Normal’  <50 and Female=’0’ ‘Low’    >=40 and Male=’1’ ‘Normal’  <40 and Male=’0’ ‘Low’ |
| LDL | Numeric | Structured test results | May confer ischemia risk. Extracted with local codes/concepts from HIE data | Missing=’1’ ‘Normal’  <100 =’1’ ‘Normal’  100-160 =’2’ ‘Baseline High’  >160 =’3’ ‘High’ |
| Triglycerides | Numeric | Structured test results | May confer ischemia risk. Extracted with local codes/concepts from HIE data | Missing=’1’ ‘Normal’  <150 =’1’ ‘Normal’  >=150=’3’ ‘High’ |

***EKG Reports***

| Variable | Variable Type | Variable Details | Notes and Logic | Coding |
| --- | --- | --- | --- | --- |
| AF | Numeric | 1=Yes, 0=No | Self-explanatory: used to define AF cases. Term mention in EKG Reports: “atrial fibrillation”, “atrial flutter” | IF AF=1 and Case_flg=0  Then Case_flg=1. |
| AVNRT | Numeric | 1=Yes, 0=No | AF may be misread as AVNRT. Term mention in EKG Reports:  “Junctional”, “AVNRT”, “AV nodal reentrant tachycardia” | Dropped due to low yield |
| Bundle Branch Block | Categorical | Left, Right | Cardiac conduction disease may precede AF. Term mention in EKG Reports:  “Left bundle branch block”, “right bundle branch block” |  |
| QRS Interval | TBD | Requires further exploration | TBD | Dropped due to low yield |
| PR Interval | TBD | Requires further exploration | TBD | Dropped due to low yield |

***Transthoracic or Transesophageal Echocardiogram Reports***

| Variable | Variable Type | Variable Details | Notes and Logic | Recoded |
| --- | --- | --- | --- | --- |
| Right Ventricular Hypertrophy | Categorical | Mild, Moderate, Severe, Unreported | Term mention in TTE/TEE reports: Mild / moderate / severe / unreported hypertrophy of right ventricle | Dropped due to low yield |
| Left Ventricular Hypertrophy | Categorical | Mild, Moderate, Severe, Unreported | Term mention in TTE/TEE reports: Mild / moderate / severe / unreported hypertrophy of left ventricle | Dropped due to low yield |
| Right Atrial Enlargement | Categorical | Mild, Moderate, Severe, Unreported | Term mention in TTE/TEE reports: Mild / moderate / severe / unreported enlargement of right atrium | Dropped due to low yield |
| Left Atrial Enlargement | Categorical | Mild, Moderate, Severe, Unreported | Term mention in TTE/TEE reports: Mild / moderate / severe / unreported enlargement of left atrium | Dropped due to low yield |
| Aortic Valve Stenosis | Categorical | Mild, Moderate, Severe, Unreported | Term mention in TTE/TEE reports: Mild / moderate / severe / unreported stenosis of aortic valve | Dropped due to low yield |
| Mitral Valve Stenosis | Categorical | Mild, Moderate, Severe, Unreported | Term mention in TTE/TEE reports: Mild / moderate / severe / unreported stenosis of mitral valve | Dropped due to low yield |
| Tricuspid Valve Stenosis | Categorical | Mild, Moderate, Severe, Unreported | Term mention in TTE/TEE reports: Mild / moderate / severe / unreported stenosis of triscupid valve | Dropped due to low yield |
| Aortic Valve Regurgitation | Categorical | Mild, Moderate, Severe, Unreported | Term mention in TTE/TEE reports: Mild / moderate / severe / unreported regurgitation of aortic valve | Dropped due to low yield |
| Mitral Valve Regurgitation | Categorical | Mild, Moderate, Severe, Unreported | Term mention in TTE/TEE reports: Mild / moderate / severe / unreported regurgitation of mitral valve | Dropped due to low yield |
| Tricuspid Valve Regurgitation | Categorical | Mild, Moderate, Severe, Unreported | Term mention in TTE/TEE reports: Mild / moderate / severe / unreported regurgitation of tricuspid valve | Dropped due to low yield |
| Thrombus | Numeric | 1=Yes, 0=No | Term mention in TTE/TEE reports: “thrombus” | Dropped due to low yield |
| Diastolic Dysfunction | Categorical | Grade 1, Grade 2, Grade 3, Grade 4 | Term mention in TTE/TEE reports:   - Grade 1=”Grade 1” or “impaired relaxation” - Grade 2=’”Grade 2” or “pseudonormal” - Grade 3=”Grade 3” or “restrictive” - Grade 4=”Grade 4” | Dropped due to low yield |
| Pulmonary Hypertension | Categorical | Mild, Moderate, Severe, Unreported | Term mention in TTE/TEE reports: Mild / moderate / severe / unreported severity pulmonary hypertension | Dropped due to low yield |

***CXR/CT/MRI Reports***

| Variable | Variable Type | Variable Details | Notes and Logic | Coding |
| --- | --- | --- | --- | --- |
| Cardiomegaly | Numeric | 1=Yes, 0=No | Term mention in chest x-ray, CT, or MRI report: “cardiomegaly” | Dropped due to low yield |
| Atrial Enlargement | Categorical | Left, Right | Term mention in chest x-ray, CT, or MRI report: “left atrial enlargement”, “right atrial enlargement” | Dropped due to low yield |
| Cephalization / pulmonary vascular congestion / pulmonary edema | Numeric | 1=Yes, 0=No | Term mention in chest x-ray, CT, or MRI report: “cephalization”, “pulmonary vascular congestion”, “pulmonary edema” | Dropped due to low yield |
| Stroke/infarct | Numeric | 1=Yes, 0=No | Term mention in head CT or brain MRI: “stroke”, “infarct” | Dropped due to low yield |

***Cardiac Stress Test Reports***

| Variable | Variable Type | Variable Details | Notes and Logic | Coding |
| --- | --- | --- | --- | --- |
| AF | Numeric | 1=Yes, 0=No | Extract rhythm noted in report during rest/stress/recovery:   - Atrial fibrillation: “AF”, “atrial fibrillation”, “a-fib”, “Afib”, “Afl”, “atrial flutter”, “A-fl” | Dropped due to low yield |
| AVNRT | Numeric | 1=Yes, 0=No | Extract rhythm noted in report during rest/stress/recovery:   - Ventricular tachycardia: "VT" , “ventricular tachycardia”, “AVNRT”, “AV nodal reentrant tachycardia”, “AVRT”, "AV reciprocating tachycardia”, "atrioventricular reciprocating tachycardia" | Dropped due to low yield |
| SVT | Numeric | 1=Yes, 0=No | Extract rhythm noted in report during rest/stress/recovery:  Supraventricular tachycardia: " SVT”, “supraventricular tachycardia” | Dropped due to low yield |

***Left Heart Catheterization/Coronary Angiography Reports***

| Variable | Variable Type | Variable Details | Notes and Logic | Recoded |
| --- | --- | --- | --- | --- |
| Coronary Vessel Stenosis | Numeric | 1=Yes, 0=No | See “ischemia” above.  Term mention in cath/angiography reports: “Stenosis” | Dropped due to low yield |
| Coronary Vessel Occlusion |  |  | See “ischemia” above.  Term mention in cath/angiography reports: “occlusion”, “occluded” | Dropped due to low yield |
| PCI | Numeric | 1=Yes, 0=No | See “ischemia” above.  Term mention in cath/angiography reports:  “Percutaneous coronary intervention”, “PCI”, “angioplasty”, “stent” | Dropped due to low yield |

Table S2. **Derived variables utilized in model development.**

***Derived Variables***

| Variable | Variable Type | Variable Details | Notes and Logic | Recoded |
| --- | --- | --- | --- | --- |
| KidneyC | Class | 1=Yes, 0=No | Created | IF BUNC=3 ‘High’ or Chronic_Kidney_disease=’1’ or Creatinine=3 ‘High’ or Endstage_renal=1 then KidneyC=1, Else KidneycC=0 |
| DiabetesC | Class | 1=Yes, 0=No | Created | If A1CC=’3’ ‘High’ or Diabetes_mellitus=1 then  DiabetesC=1, Else DiabetesC=0 |
| Heart Disease | Class | 1=Yes, 0=No | Created | IF Troponin=’3’ ‘High’ or Heart_attack=’1’ then HDC=1 Else HDC=0 |
| LipidC | Class | 1=Yes, 0=No | Created | IF CholesterolC=’3’ ‘High’ or LDLC=’3’ ‘High’ then LipidC=1 ELSE LipidC=0 |
| LiverC | Class | 1=Yes, 0=No | Created | IF ALT=’3’ ‘High’ or ASTC=’3’ ‘HIGH’ then LIVERC=’1’ ELSE LIVERC=’0’ |
| Treated Hypertension | Class | 1=Yes, 0=No | Created | IF Hypertension=1 and Hypertension Meds=1 then  HTN_TRT=1, ELSE, HTN_TRT=0 |

Table S3. **Laboratory values in model development data set.**

| **Variable** | **Overall**  **N=53552** | **AF**  **N=31474** | **No-AF**  **N=22078** | **P-value** |
| --- | --- | --- | --- | --- |
| Creatinine (mg/dL) |  |  |  | <.0001 |
| - High | 6016 (11.2%) | 4777 (15.2%) | 1239 (5.6%) |  |
| - Low | 933 (1.7%) | 646 (2.1%) | 287 (1.3%) |  |
| - Normal | 46603 (87.0%) | 26051 (82.8%) | 20552 (93.1%) |  |
| Max Creatinine (mg/dL) |  |  |  | <.0001 |
| - High | 7883 (14.7%) | 6208 (19.7%) | 1675 (7.6%) |  |
| - Low | 726 (1.4%) | 510 (1.6%) | 216 (1.0%) |  |
| - Normal | 44943 (83.9%) | 24756 (78.7%) | 20187 (91.4%) |  |
| Min Creatinine (mg/dL) |  |  |  | <.0001 |
| - High | 3663 (6.8%) | 2896 (9.2%) | 767 (3.5%) |  |
| - Low | 2083 (3.9%) | 1478 (4.7%) | 605 (2.7%) |  |
| - Normal | 47806 (89.3%) | 27100 (86.1%) | 20706 (93.8%) |  |
| Potassium (mEq/L) |  |  |  | <.0001 |
| - High | 493 (0.9%) | 347 (1.1%) | 146 (0.7%) |  |
| - Low | 1382 (2.6%) | 874 (2.8%) | 508 (2.3%) |  |
| - Normal | 51677 (96.5%) | 30253 (96.1%) | 21424 (97.0%) |  |
| Max Potassium (mEq/L) |  |  |  | <.0001 |
| - High | 1948 (3.6%) | 1517 (4.8%) | 431 (2.0%) |  |
| - Low | 468 (0.9%) | 242 (0.8%) | 226 (1.0%) |  |
| - Normal | 51136 (95.5%) | 29715 (94.4%) | 21421 (97.0%) |  |
| Min Potassium (mEq/L) |  |  |  | <.0001 |
| - High | 149 (0.3%) | 90 (0.3%) | 59 (0.3%) |  |
| - Low | 5705 (10.7%) | 4090 (13.0%) | 1615 (7.3%) |  |
| - Normal | 47698 (89.1%) | 27294 (86.7%) | 20404 (92.4%) |  |
| Sodium (mEq/L) |  |  |  | <.0001 |
| - Low | 5197 (9.7%) | 3720 (11.8%) | 1477 (6.7%) |  |
| - Normal | 48355 (90.3%) | 27754 (88.2%) | 20601 (93.3%) |  |
| Max Sodium (mEq/L) |  |  |  | <.0001 |
| - Low | 1950 (3.6%) | 1293 (4.1%) | 657 (3.0%) |  |
| - Normal | 51602 (96.4%) | 30181 (95.9%) | 21421 (97.0%) |  |
| Min Sodium (mEq/L) |  |  |  | <.0001 |
| - Low | 11628 (21.7%) | 8586 (27.3%) | 3042 (13.8%) |  |
| - Normal | 41924 (78.3%) | 22888 (72.7%) | 19036 (86.2%) |  |
| Calcium (mg/dL) |  |  |  | <.0001 |
| - High | 472 (0.9%) | 302 (1.0%) | 170 (0.8%) |  |
| - Low | 2939 (5.5%) | 2168 (6.9%) | 771 (3.5%) |  |
| - Normal | 50141 (93.6%) | 29004 (92.2%) | 21137 (95.7%) |  |
| Hemoglobin (g/dL) |  |  |  | <.0001 |
| - High | 183 (0.3%) | 99 (0.3%) | 84 (0.4%) |  |
| - Low | 13112 (24.5%) | 9819 (31.2%) | 3293 (14.9%) |  |
| - Normal | 40257 (75.2%) | 21556 (68.5%) | 18701 (84.7%) |  |
| Max Hemoglobin (g/dL) |  |  |  | <.0001 |
| - High | 433 (0.8%) | 273 (0.9%) | 160 (0.7%) |  |
| - Low | 8216 (15.3%) | 6276 (19.9%) | 1940 (8.8%) |  |
| - Normal | 44903 (83.8%) | 24925 (79.2%) | 19978 (90.5%) |  |
| MIN Hemoglobin (g/dL) |  |  |  | <.0001 |
| - High | 106 (0.2%) | 57 (0.2%) | 49 (0.2%) |  |
| - Low | 16397 (30.6%) | 12119 (38.5%) | 4278 (19.4%) |  |
| - Normal | 37049 (69.2%) | 19298 (61.3%) | 17751 (80.4%) |  |
| Hematocrit (%) |  |  |  | <.0001 |
| - High | 546 (1.0%) | 348 (1.1%) | 198 (0.9%) |  |
| - Low | 13566 (25.3%) | 10010 (31.8%) | 3556 (16.1%) |  |
| - Normal | 39440 (73.6%) | 21116 (67.1%) | 18324 (83.0%) |  |
| Max Hematocrit (%) |  |  |  | <.0001 |
| - High | 1180 (2.2%) | 799 (2.5%) | 381 (1.7%) |  |
| - Low | 8731 (16.3%) | 6522 (20.7%) | 2209 (10.0%) |  |
| - Normal | 43641 (81.5%) | 24153 (76.7%) | 19488 (88.3%) |  |
| Min Hematocrit (%) |  |  |  | <.0001 |
| - High | 318 (0.6%) | 193 (0.6%) | 125 (0.6%) |  |
| - Low | 17300 (32.3%) | 12609 (40.1%) | 4691 (21.2%) |  |
| - Normal | 35934 (67.1%) | 18672 (59.3%) | 17262 (78.2%) |  |
| Platelets (µL) |  |  |  | <.0001 |
| - Low | 3633 (6.8%) | 2857 (9.1%) | 776 (3.5%) |  |
| - Normal | 49919 (93.2%) | 28617 (90.9%) | 21302 (96.5%) |  |
| ALT (U/L) |  |  |  | <.0001 |
| - High | 703 (1.3%) | 356 (1.1%) | 347 (1.6%) |  |
| - Low | 2468 (4.6%) | 1853 (5.9%) | 615 (2.8%) |  |
| - Normal | 50381 (94.1%) | 29265 (93.0%) | 21116 (95.6%) |  |
| Neutrophils (%) |  |  |  | <.0001 |
| - High | 4081 (7.6%) | 2950 (9.4%) | 1131 (5.1%) |  |
| - Low | 16885 (31.5%) | 10684 (33.9%) | 6201 (28.1%) |  |
| - Normal | 32586 (60.8%) | 17840 (56.7%) | 14746 (66.8%) |  |
| AST (U/L) |  |  |  | <.0001 |
| - Low | 308 (0.6%) | 223 (0.7%) | 85 (0.4%) |  |
| - Normal | 53244 (99.4%) | 31251 (99.3%) | 21993 (99.6%) |  |
| Albumin (g/dL) |  |  |  | <.0001 |
| - Low | 4110 (7.7%) | 3264 (10.4%) | 846 (3.8%) |  |
| - Normal | 49442 (92.3%) | 28210 (89.6%) | 21232 (96.2%) |  |
| Alkaline PhosphateC (U/L) |  |  |  | <.0001 |
| - High | 1025 (1.9%) | 669 (2.1%) | 356 (1.6%) |  |
| - Low | 152 (0.3%) | 104 (0.3%) | 48 (0.2%) |  |
| - Normal | 52375 (97.8%) | 30701 (97.5%) | 21674 (98.2%) |  |
| LDL (mg/dL) |  |  |  | <.0001 |
| - BL_High | 9391 (17.5%) | 4861 (15.4%) | 4530 (20.5%) |  |
| - High | 122 (0.2%) | 60 (0.2%) | 62 (0.3%) |  |
| - Normal | 44039 (82.2%) | 26553 (84.4%) | 17486 (79.2%) |  |
| Bilirubin (mg/dL) |  |  |  | 0.1752 |
| - Low | 1703 (3.2%) | 1028 (3.3%) | 675 (3.1%) |  |
| - Normal | 51849 (96.8%) | 30446 (96.7%) | 21403 (96.9%) |  |
| Cholesterol (mg/dL) |  |  |  | <.0001 |
| - High | 34917 (65.2%) | 19471 (61.9%) | 15446 (70.0%) |  |
| - Normal | 18635 (34.8%) | 12003 (38.1%) | 6632 (30.0%) |  |
| HDL (mg/dL) |  |  |  | <.0001 |
| - Low | 11085 (20.7%) | 7001 (22.2%) | 4084 (18.5%) |  |
| - Normal | 42467 (79.3%) | 24473 (77.8%) | 17994 (81.5%) |  |
| Triglycerides (mg/dL) |  |  |  | 0.8843 |
| - High | 6628 (12.4%) | 3890 (12.4%) | 2738 (12.4%) |  |
| - Normal | 46924 (87.6%) | 27584 (87.6%) | 19340 (87.6%) |  |
| Lymphocytes (%) |  |  |  | <.0001 |
| - Low | 11859 (22.1%) | 8258 (26.2%) | 3601 (16.3%) |  |
| - Normal | 41693 (77.9%) | 23216 (73.8%) | 18477 (83.7%) |  |
| Thyroid Stimulating Hormone (µU/mL) |  |  |  | <.0001 |
| - High | 669 (1.2%) | 468 (1.5%) | 201 (0.9%) |  |
| - Low | 1269 (2.4%) | 786 (2.5%) | 483 (2.2%) |  |
| - Normal | 51614 (96.4%) | 30220 (96.0%) | 21394 (96.9%) |  |
| A1c (%) |  |  |  | <.0001 |
| - BL_High | 4536 (8.5%) | 3018 (9.6%) | 1518 (6.9%) |  |
| - High | 5361 (10.0%) | 3794 (12.1%) | 1567 (7.1%) |  |
| - Normal | 43655 (81.5%) | 24662 (78.4%) | 18993 (86.0%) |  |
| Troponin (ng/mL) |  |  |  | <.0001 |
| - High | 1456 (2.7%) | 1141 (3.6%) | 315 (1.4%) |  |
| - Normal | 52096 (97.3%) | 30333 (96.4%) | 21763 (98.6%) |  |
| Max Troponin (ng/mL) |  |  |  | <.0001 |
| - High | 2020 (3.8%) | 1612 (5.1%) | 408 (1.8%) |  |
| - Normal | 51532 (96.2%) | 29862 (94.9%) | 21670 (98.2%) |  |
| Min Troponin (ng/mL) |  |  |  | <.0001 |
| - High | 1043 (1.9%) | 777 (2.5%) | 266 (1.2%) |  |
| - Normal | 52509 (98.1%) | 30697 (97.5%) | 21812 (98.8%) |  |
| Magnesium (mEq/L) |  |  |  | <.0001 |
| - Low | 490 (0.9%) | 375 (1.2%) | 115 (0.5%) |  |
| - Normal | 53062 (99.1%) | 31099 (98.8%) | 21963 (99.5%) |  |
| Parathyroid Hormone (pg/mL) |  |  |  | <.0001 |
| - High | 735 (1.4%) | 601 (1.9%) | 134 (0.6%) |  |
| - Low | 24 (0.0%) | 19 (0.1%) | 5 (0.0%) |  |
| - Normal | 52793 (98.6%) | 30854 (98.0%) | 21939 (99.4%) |  |

Table S4. **Comorbidities in model development data set.**

| **Variables** | **Overall**  **N=53552** | **AFIB**  **N=31474** | **No-AFIB**  **N=22078** | **P-value** |
| --- | --- | --- | --- | --- |
| Body Mass Index |  |  |  | <.0001 |
| - Missing | 33926 (63.4%) | 18468 (58.7%) | 15458 (70.0%) |  |
| - Normal weight:18.5 to 24.9 | 4008 (7.5%) | 2684 (8.5%) | 1324 (6.0%) |  |
| - Obese: >=30 | 9258 (17.3%) | 6139 (19.5%) | 3119 (14.1%) |  |
| - Overweight: 25 to 29.9 | 6013 (11.2%) | 3926 (12.5%) | 2087 (9.5%) |  |
| - Underweight: <18.5 | 347 (0.6%) | 257 (0.8%) | 90 (0.4%) |  |
| IMMUNE_INFLAM_DISEASE |  |  |  | 0.0003 |
| - No | 47723 (89.1%) | 27919 (88.7%) | 19804 (89.7%) |  |
| - Yes | 5829 (10.9%) | 3555 (11.3%) | 2274 (10.3%) |  |
| CHRONIC_ISCHEMIC_HEART_DISEASE |  |  |  | <.0001 |
| - No | 46776 (87.3%) | 25831 (82.1%) | 20945 (94.9%) |  |
| - Yes | 6776 (12.7%) | 5643 (17.9%) | 1133 (5.1%) |  |
| HYPERTENSION |  |  |  | <.0001 |
| - No | 31141 (58.2%) | 15227 (48.4%) | 15914 (72.1%) |  |
| - Yes | 22411 (41.8%) | 16247 (51.6%) | 6164 (27.9%) |  |
| SHOCK |  |  |  | <.0001 |
| - No | 50187 (93.7%) | 28881 (91.8%) | 21306 (96.5%) |  |
| - Yes | 3365 (6.3%) | 2593 (8.2%) | 772 (3.5%) |  |
| CHRONIC_KIDNEY_DISEASE |  |  |  | <.0001 |
| - No | 48804 (91.1%) | 27436 (87.2%) | 21368 (96.8%) |  |
| - Yes | 4748 (8.9%) | 4038 (12.8%) | 710 (3.2%) |  |
| END_STAGE_RENAL_DISEASE |  |  |  | <.0001 |
| - No | 52832 (98.7%) | 30834 (98.0%) | 21998 (99.6%) |  |
| - Yes | 720 (1.3%) | 640 (2.0%) | 80 (0.4%) |  |
| Diabetes |  |  |  | <.0001 |
| - No | 42479 (79.3%) | 23288 (74.0%) | 19191 (86.9%) |  |
| - Yes | 11073 (20.7%) | 8186 (26.0%) | 2887 (13.1%) |  |
| OSTEOARTHRITIS |  |  |  | <.0001 |
| - No | 47619 (88.9%) | 27218 (86.5%) | 20401 (92.4%) |  |
| - Yes | 5933 (11.1%) | 4256 (13.5%) | 1677 (7.6%) |  |
| CHRONIC_RESPIRATORY_FAILURE |  |  |  | <.0001 |
| - No | 51627 (96.4%) | 29864 (94.9%) | 21763 (98.6%) |  |
| - Yes | 1925 (3.6%) | 1610 (5.1%) | 315 (1.4%) |  |
| Chronic Obstructive Pulmonary Disease |  |  |  | <.0001 |
| - No | 45661 (85.3%) | 25565 (81.2%) | 20096 (91.0%) |  |
| - Yes | 7891 (14.7%) | 5909 (18.8%) | 1982 (9.0%) |  |
| PERIPHERAL_VASCULAR_DISEASE |  |  |  | <.0001 |
| - No | 49091 (91.7%) | 27793 (88.3%) | 21298 (96.5%) |  |
| - Yes | 4461 (8.3%) | 3681 (11.7%) | 780 (3.5%) |  |
| SLEEP_APNEA |  |  |  | <.0001 |
| - No | 50297 (93.9%) | 29066 (92.3%) | 21231 (96.2%) |  |
| - Yes | 3255 (6.1%) | 2408 (7.7%) | 847 (3.8%) |  |
| HEART_ATTACK |  |  |  | <.0001 |
| - No | 44642 (83.4%) | 24222 (77.0%) | 20420 (92.5%) |  |
| - Yes | 8910 (16.6%) | 7252 (23.0%) | 1658 (7.5%) |  |
| HEART_FAILURE |  |  |  | <.0001 |

**Table S5: Model performance in development and validation sets**

|  | **C-statistic (95% confidence interval)** | |
| --- | --- | --- |
| **Model** | *Development set* | *Validation set* |
| UNAFIED model | 0.7956 (0.7917--0.7994) | 0.8061 (0.802--0.8102) |
| Free-for-all 10-variable model | 0.7959 (0.7921--0.7997) | 0.8048 (0.8007--0.8089) |
| Parsimonious 5-variable model | 0.7851 (0.7812--0.789) | 0.7866 (0.7823--0.7908) |
| Aronson model | 0.7915 (0.7877--0.7954) | 0.7935 (0.7893--0.7977) |
| Volgman model | 0.7777 (0.7738--0.7817) | 0.7796 (0.7753--0.7838) |

**Table S6. CHA₂DS₂-VASc descriptive statistics among UNAFIED and non-UNAFIED patients**

|  | **CHA₂DS₂-VASc Score** | **UNAFIED patients** | Non-UNAFIED patients | **All** |
| --- | --- | --- | --- | --- |
| **CHA₂DS₂-VASc, mean (S.D.)** |  | 1.694 (1.69) | 1.213 (1.40) | 2.546 (1.79) |
|  | <2 | 2334  (29.48%) | 8331  (58.03%) | 10665 (47.89%) |
|  | >=2 | 5582 (70.52%) | 6025 (41.97%) | 11607 (52.11%) |
| By age |  |  |  |  |
| <65 | <2 | 1421 (40.10%) | 8105 (60.52%) | 9526 (56.25%) |
|  | >=2 | 2122 (59.90%) | 5288 (39.48%) | 7410 (43.75%) |
| 65+ | <2 | 913 (20.88%) | 226 (23.47%) | 1139 (21.35%) |
|  | >=2 | 3460 (79.12%) | 737 (76.53%) | 4197 (78.65%) |
| ***By sex (percent within sex)*** |  |  |  |  |
| Female | <2 | 669 (17.02%) | 4353 (30.32%) | 5022 (38.50%) |
|  | >=2 | 3261 (82.98%) | 4760 (33.16%) | 8021 (61.50%) |
| Male | <2 | 1665 (41.77%) | 3978 (75.87%) | 5643 (61.14%) |
|  | >=2 | 2321 (58.23%) | 1265 (24.13%) | 3586 (38.86%) |
| ***By Age and sex (percent within sex)*** |  |  |  |  |
| <65 Female | <2 | 206 (15.91%) | 4184 (49.68%) | 4390 (45.18%) |
|  | >=2 | 1089 (84.09%) | 4238 (50.32%) | 5327 (54.82%) |
| <65 Male | <2 | 1215 (54.05%) | 3921 (78.88%) | 5136 (71.15%) |
|  | >=2 | 1033 (45.95%) | 1050 (21.12%) | 2083 (28.85%) |
| 65+ Female | <2 | 463 (17.57%) | 169 (24.46%) | 632 (19.00%) |
|  | >=2 | 2172 (82.43%) | 522 (75.54%) | 2694 (81.00%) |
| 65+ Male | <2 | 450 (25.89%) | 57 (20.96%) | 507 (25.22%) |
|  | >=2 | 1288 (74.11%) | 215 (79.04%) | 1503 (74.78%) |

**Table S7. Laboratory values in model validation data set.**

Note: Values expressed as n(%), mean±standard deviation or median (Q1,Q3)

Note: P-value comparisons across case_flg categories are based on Chi-square test (or Fisher's Exact test) for categorical variables; p-values for continuous variables are based on ANOVA or Wilcoxon (Normal Approximation)

| **variable label** | **Overall N=44772** | **AFIB N=26476** | **No-AFIB N=18296** | **P-value** |
| --- | --- | --- | --- | --- |
|  |  |  |  |  |
| Creatinine (mg/dL) |  |  |  | <.0001 |
| - High | 5482 (12.2%) | 4374 (16.5%) | 1108 (6.1%) |  |
| - Low | 722 (1.6%) | 513 (1.9%) | 209 (1.1%) |  |
| - Normal | 38568 (86.1%) | 21589 (81.5%) | 16979 (92.8%) |  |
| Max Creatinine (mg/dL) |  |  |  | <.0001 |
| - High | 7499 (16.7%) | 5917 (22.3%) | 1582 (8.6%) |  |
| - Low | 562 (1.3%) | 396 (1.5%) | 166 (0.9%) |  |
| - Normal | 36711 (82.0%) | 20163 (76.2%) | 16548 (90.4%) |  |
| Min Creatinine (mg/dL) |  |  |  | <.0001 |
| - High | 3124 (7.0%) | 2469 (9.3%) | 655 (3.6%) |  |
| - Low | 1816 (4.1%) | 1345 (5.1%) | 471 (2.6%) |  |
| - Normal | 39832 (89.0%) | 22662 (85.6%) | 17170 (93.8%) |  |
| Potassium (mEq/L) |  |  |  | <.0001 |
| - Low | 1403 (3.1%) | 925 (3.5%) | 478 (2.6%) |  |
| - Normal | 43369 (96.9%) | 25551 (96.5%) | 17818 (97.4%) |  |
| Max Potassium (mEq/L) |  |  |  | 0.0875 |
| - Low | 367 (0.8%) | 201 (0.8%) | 166 (0.9%) |  |
| - Normal | 44405 (99.2%) | 26275 (99.2%) | 18130 (99.1%) |  |
| Min Potassium (mEq/L) |  |  |  | <.0001 |
| - Low | 6063 (13.5%) | 4488 (17.0%) | 1575 (8.6%) |  |
| - Normal | 38709 (86.5%) | 21988 (83.0%) | 16721 (91.4%) |  |
| Sodium (mEq/L) |  |  |  | <.0001 |
| - Low | 4102 (9.2%) | 3014 (11.4%) | 1088 (5.9%) |  |
| - Normal | 40670 (90.8%) | 23462 (88.6%) | 17208 (94.1%) |  |
| Max Sodium (mEq/L) |  |  |  | <.0001 |
| - Low | 1309 (2.9%) | 910 (3.4%) | 399 (2.2%) |  |
| - Normal | 43463 (97.1%) | 25566 (96.6%) | 17897 (97.8%) |  |
| Min Sodium (mEq/L) |  |  |  | <.0001 |
| - Low | 10288 (23.0%) | 7778 (29.4%) | 2510 (13.7%) |  |
| - Normal | 34484 (77.0%) | 18698 (70.6%) | 15786 (86.3%) |  |
| Calcium (mg/dL) |  |  |  | <.0001 |
| - High | 614 (1.4%) | 372 (1.4%) | 242 (1.3%) |  |
| - Low | 3156 (7.0%) | 2367 (8.9%) | 789 (4.3%) |  |
| - Normal | 41002 (91.6%) | 23737 (89.7%) | 17265 (94.4%) |  |
| Hemoglobin (g/dL) |  |  |  | <.0001 |
| - High | 145 (0.3%) | 98 (0.4%) | 47 (0.3%) |  |
| - Low | 12329 (27.5%) | 9357 (35.3%) | 2972 (16.2%) |  |
| - Normal | 32298 (72.1%) | 17021 (64.3%) | 15277 (83.5%) |  |
| Max Hemoglobin (g/dL) |  |  |  | <.0001 |
| - High | 396 (0.9%) | 267 (1.0%) | 129 (0.7%) |  |
| - Low | 6932 (15.5%) | 5366 (20.3%) | 1566 (8.6%) |  |
| - Normal | 37444 (83.6%) | 20843 (78.7%) | 16601 (90.7%) |  |
| MIN Hemoglobin (g/dL) |  |  |  | <.0001 |
| - High | 67 (0.1%) | 45 (0.2%) | 22 (0.1%) |  |
| - Low | 15831 (35.4%) | 11811 (44.6%) | 4020 (22.0%) |  |
| - Normal | 28874 (64.5%) | 14620 (55.2%) | 14254 (77.9%) |  |
| Hematocrit (%) |  |  |  | <.0001 |
| - High | 291 (0.6%) | 198 (0.7%) | 93 (0.5%) |  |
| - Low | 13547 (30.3%) | 9982 (37.7%) | 3565 (19.5%) |  |
| - Normal | 30934 (69.1%) | 16296 (61.6%) | 14638 (80.0%) |  |
| Max Hematocrit (%) |  |  |  | <.0001 |
| - High | 793 (1.8%) | 545 (2.1%) | 248 (1.4%) |  |
| - Low | 7964 (17.8%) | 5912 (22.3%) | 2052 (11.2%) |  |
| - Normal | 36015 (80.4%) | 20019 (75.6%) | 15996 (87.4%) |  |
| Min Hematocrit (%) |  |  |  | <.0001 |
| - High | 131 (0.3%) | 86 (0.3%) | 45 (0.2%) |  |
| - Low | 17314 (38.7%) | 12564 (47.5%) | 4750 (26.0%) |  |
| - Normal | 27327 (61.0%) | 13826 (52.2%) | 13501 (73.8%) |  |
| Platelets (µL) |  |  |  | <.0001 |
| - Low | 3280 (7.3%) | 2558 (9.7%) | 722 (3.9%) |  |
| - Normal | 41492 (92.7%) | 23918 (90.3%) | 17574 (96.1%) |  |
| ALT (U/L) |  |  |  | <.0001 |
| - High | 1206 (2.7%) | 651 (2.5%) | 555 (3.0%) |  |
| - Low | 1636 (3.7%) | 1251 (4.7%) | 385 (2.1%) |  |
| - Normal | 41930 (93.7%) | 24574 (92.8%) | 17356 (94.9%) |  |
| Neutrophils (%) |  |  |  | <.0001 |
| - High | 4152 (9.3%) | 3076 (11.6%) | 1076 (5.9%) |  |
| - Low | 14211 (31.7%) | 9038 (34.1%) | 5173 (28.3%) |  |
| - Normal | 26409 (59.0%) | 14362 (54.2%) | 12047 (65.8%) |  |
| AST (U/L) |  |  |  | <.0001 |
| - Low | 201 (0.4%) | 150 (0.6%) | 51 (0.3%) |  |
| - Normal | 44571 (99.6%) | 26326 (99.4%) | 18245 (99.7%) |  |
| Albumin (g/dL) |  |  |  | <.0001 |
| - Low | 4361 (9.7%) | 3530 (13.3%) | 831 (4.5%) |  |
| - Normal | 40411 (90.3%) | 22946 (86.7%) | 17465 (95.5%) |  |
| Alkaline PhosphateC (U/L) |  |  |  | <.0001 |
| - High | 1159 (2.6%) | 811 (3.1%) | 348 (1.9%) |  |
| - Low | 103 (0.2%) | 69 (0.3%) | 34 (0.2%) |  |
| - Normal | 43510 (97.2%) | 25596 (96.7%) | 17914 (97.9%) |  |
| LDL (mg/dL) |  |  |  | <.0001 |
| - BL_High | 8418 (18.8%) | 4432 (16.7%) | 3986 (21.8%) |  |
| - High | 155 (0.3%) | 67 (0.3%) | 88 (0.5%) |  |
| - Normal | 36199 (80.9%) | 21977 (83.0%) | 14222 (77.7%) |  |
| Bilirubin (mg/dL) |  |  |  | <.0001 |
| - High | 520 (1.2%) | 348 (1.3%) | 172 (0.9%) |  |
| - Low | 1593 (3.6%) | 1043 (3.9%) | 550 (3.0%) |  |
| - Normal | 42659 (95.3%) | 25085 (94.7%) | 17574 (96.1%) |  |
| Cholesterol (mg/dL) |  |  |  | <.0001 |
| - High | 28919 (64.6%) | 16092 (60.8%) | 12827 (70.1%) |  |
| - Normal | 15853 (35.4%) | 10384 (39.2%) | 5469 (29.9%) |  |
| HDL (mg/dL) |  |  |  | <.0001 |
| - Low | 9432 (21.1%) | 6191 (23.4%) | 3241 (17.7%) |  |
| - Normal | 35340 (78.9%) | 20285 (76.6%) | 15055 (82.3%) |  |
| Triglycerides (mg/dL) |  |  |  | <.0001 |
| - High | 5428 (12.1%) | 3369 (12.7%) | 2059 (11.3%) |  |
| - Normal | 39344 (87.9%) | 23107 (87.3%) | 16237 (88.7%) |  |
| Lymphocytes (%) |  |  |  | <.0001 |
| - Low | 13141 (29.4%) | 9248 (34.9%) | 3893 (21.3%) |  |
| - Normal | 31631 (70.6%) | 17228 (65.1%) | 14403 (78.7%) |  |
| Thryoid Stimulating Hormone (µU/mL) |  |  |  | <.0001 |
| - High | 647 (1.4%) | 441 (1.7%) | 206 (1.1%) |  |
| - Low | 1194 (2.7%) | 781 (2.9%) | 413 (2.3%) |  |
| - Normal | 42931 (95.9%) | 25254 (95.4%) | 17677 (96.6%) |  |
| A1c (%) |  |  |  | <.0001 |
| - BL_High | 4217 (9.4%) | 2908 (11.0%) | 1309 (7.2%) |  |
| - High | 5107 (11.4%) | 3754 (14.2%) | 1353 (7.4%) |  |
| - Normal | 35448 (79.2%) | 19814 (74.8%) | 15634 (85.5%) |  |
| Troponin (ng/mL) |  |  |  | <.0001 |
| - High | 3191 (7.1%) | 2427 (9.2%) | 764 (4.2%) |  |
| - Normal | 41581 (92.9%) | 24049 (90.8%) | 17532 (95.8%) |  |
| Max Troponin (ng/mL) |  |  |  | <.0001 |
| - High | 4062 (9.1%) | 3180 (12.0%) | 882 (4.8%) |  |
| - Normal | 40710 (90.9%) | 23296 (88.0%) | 17414 (95.2%) |  |
| Min Troponin (ng/mL) |  |  |  | <.0001 |
| - High | 2351 (5.3%) | 1702 (6.4%) | 649 (3.5%) |  |
| - Normal | 42421 (94.7%) | 24774 (93.6%) | 17647 (96.5%) |  |
| Magnesium (mEq/L) |  |  |  | <.0001 |
| - Low | 515 (1.2%) | 412 (1.6%) | 103 (0.6%) |  |
| - Normal | 44257 (98.8%) | 26064 (98.4%) | 18193 (99.4%) |  |
| Parathyroid Hormone (pg/mL) |  |  |  | <.0001 |
| - High | 603 (1.3%) | 493 (1.9%) | 110 (0.6%) |  |
| - Low | 26 (0.1%) | 19 (0.1%) | 7 (0.0%) |  |
| - Normal | 44143 (98.6%) | 25964 (98.1%) | 18179 (99.4%) |  |

**Table S8. Comorbidities in model validation data set.**

Note: Values expressed as n(%), mean±standard deviation or median (Q1,Q3)

Note: P-value comparisons across case_flg categories are based on Chi-square test (or Fisher's Exact test) for categorical variables; p-values for continuous variables are based on ANOVA or Wilcoxon (Normal Approximation)

| **variable label** | **Overall N=44772** | **AFIB N=26476** | **No-AFIB N=18296** | **P-value** |
| --- | --- | --- | --- | --- |
|  |  |  |  |  |
| Body Mass Index |  |  |  | <.0001 |
| - Missing | 32060 (71.6%) | 18074 (68.3%) | 13986 (76.4%) |  |
| - Normal weight:18.5 to 24.9 | 2801 (6.3%) | 1843 (7.0%) | 958 (5.2%) |  |
| - Obese: >=30 | 5890 (13.2%) | 3945 (14.9%) | 1945 (10.6%) |  |
| - Overweight: 25 to 29.9 | 3797 (8.5%) | 2438 (9.2%) | 1359 (7.4%) |  |
| - Underweight: <18.5 | 224 (0.5%) | 176 (0.7%) | 48 (0.3%) |  |
| IMMUNE_INFLAM_DISEASE |  |  |  | <.0001 |
| - No | 40356 (90.1%) | 23674 (89.4%) | 16682 (91.2%) |  |
| - Yes | 4416 (9.9%) | 2802 (10.6%) | 1614 (8.8%) |  |
| CHRONIC_ISCHEMIC_HEART_DISEASE |  |  |  | <.0001 |
| - No | 40025 (89.4%) | 22458 (84.8%) | 17567 (96.0%) |  |
| - Yes | 4747 (10.6%) | 4018 (15.2%) | 729 (4.0%) |  |
| HYPERTENSION |  |  |  | <.0001 |
| - No | 29325 (65.5%) | 15071 (56.9%) | 14254 (77.9%) |  |
| - Yes | 15447 (34.5%) | 11405 (43.1%) | 4042 (22.1%) |  |
| SHOCK |  |  |  | <.0001 |
| - No | 42278 (94.4%) | 24526 (92.6%) | 17752 (97.0%) |  |
| - Yes | 2494 (5.6%) | 1950 (7.4%) | 544 (3.0%) |  |
| CHRONIC_KIDNEY_DISEASE |  |  |  | <.0001 |
| - No | 41811 (93.4%) | 23897 (90.3%) | 17914 (97.9%) |  |
| - Yes | 2961 (6.6%) | 2579 (9.7%) | 382 (2.1%) |  |
| END_STAGE_RENAL_DISEASE |  |  |  | <.0001 |
| - No | 44197 (98.7%) | 25957 (98.0%) | 18240 (99.7%) |  |
| - Yes | 575 (1.3%) | 519 (2.0%) | 56 (0.3%) |  |
| Diabetes |  |  |  | <.0001 |
| - No | 36940 (82.5%) | 20576 (77.7%) | 16364 (89.4%) |  |
| - Yes | 7832 (17.5%) | 5900 (22.3%) | 1932 (10.6%) |  |
| OSTEOARTHRITIS |  |  |  | <.0001 |
| - No | 41079 (91.8%) | 23816 (90.0%) | 17263 (94.4%) |  |
| - Yes | 3693 (8.2%) | 2660 (10.0%) | 1033 (5.6%) |  |
| CHRONIC_RESPIRATORY_FAILURE |  |  |  | <.0001 |
| - No | 42900 (95.8%) | 24921 (94.1%) | 17979 (98.3%) |  |
| - Yes | 1872 (4.2%) | 1555 (5.9%) | 317 (1.7%) |  |
| Chronic Obstructive Pulmonary Disease |  |  |  | <.0001 |
| - No | 38567 (86.1%) | 21666 (81.8%) | 16901 (92.4%) |  |
| - Yes | 6205 (13.9%) | 4810 (18.2%) | 1395 (7.6%) |  |
| PERIPHERAL_VASCULAR_DISEASE |  |  |  | <.0001 |
| - No | 41772 (93.3%) | 24043 (90.8%) | 17729 (96.9%) |  |
| - Yes | 3000 (6.7%) | 2433 (9.2%) | 567 (3.1%) |  |
| SLEEP_APNEA |  |  |  | <.0001 |
| - No | 42732 (95.4%) | 24947 (94.2%) | 17785 (97.2%) |  |
| - Yes | 2040 (4.6%) | 1529 (5.8%) | 511 (2.8%) |  |
| HEART_ATTACK |  |  |  | <.0001 |
| - No | 38456 (85.9%) | 21204 (80.1%) | 17252 (94.3%) |  |
| - Yes | 6316 (14.1%) | 5272 (19.9%) | 1044 (5.7%) |  |
| HEART_FAILURE |  |  |  | <.0001 |
| - No | 41582 (92.9%) | 23585 (89.1%) | 17997 (98.4%) |  |
| - Yes | 3190 (7.1%) | 2891 (10.9%) | 299 (1.6%) |  |
| HOLIDAY_HEART |  |  |  |  |
| - No | 44772 (100.0%) | 26476 (100.0%) | 18296 (100.0%) |  |
| 2 or More ED Visits |  |  |  | <.0001 |
| - No | 31878 (71.2%) | 17979 (67.9%) | 13899 (76.0%) |  |
| - Yes | 12894 (28.8%) | 8497 (32.1%) | 4397 (24.0%) |  |
| HYPERTENSION_MEDS |  |  |  | <.0001 |
| - No | 33596 (75.0%) | 17935 (67.7%) | 15661 (85.6%) |  |
| - Yes | 11176 (25.0%) | 8541 (32.3%) | 2635 (14.4%) |  |
| ALCOHOL_USE |  |  |  | 0.0080 |
| - No | 44030 (98.3%) | 26002 (98.2%) | 18028 (98.5%) |  |
| - Yes | 742 (1.7%) | 474 (1.8%) | 268 (1.5%) |  |
| TOBACCO_USE |  |  |  | <.0001 |
| - No | 40050 (89.5%) | 23236 (87.8%) | 16814 (91.9%) |  |
| - Yes | 4722 (10.5%) | 3240 (12.2%) | 1482 (8.1%) |  |
| CARDIOMEGALY |  |  |  | <.0001 |
| - No | 44245 (98.8%) | 26008 (98.2%) | 18237 (99.7%) |  |
| - Yes | 527 (1.2%) | 468 (1.8%) | 59 (0.3%) |  |
| ECHO_FLG |  |  |  | 0.0677 |
| - No | 40932 (91.4%) | 24152 (91.2%) | 16780 (91.7%) |  |
| - Yes | 3840 (8.6%) | 2324 (8.8%) | 1516 (8.3%) |  |
| Kidney Disease |  |  |  | <.0001 |
| - No | 41784 (93.3%) | 23878 (90.2%) | 17906 (97.9%) |  |
| - Yes | 2988 (6.7%) | 2598 (9.8%) | 390 (2.1%) |  |
| Diabetic |  |  |  | <.0001 |
| - No | 35089 (78.4%) | 19323 (73.0%) | 15766 (86.2%) |  |
| - Yes | 9683 (21.6%) | 7153 (27.0%) | 2530 (13.8%) |  |
| Acute Heart Disease |  |  |  | <.0001 |
| - No | 36341 (81.2%) | 19709 (74.4%) | 16632 (90.9%) |  |
| - Yes | 8431 (18.8%) | 6767 (25.6%) | 1664 (9.1%) |  |
| LIPIDC |  |  |  | <.0001 |
| - No | 15847 (35.4%) | 10382 (39.2%) | 5465 (29.9%) |  |
| - Yes | 28925 (64.6%) | 16094 (60.8%) | 12831 (70.1%) |  |
| ANEMIAC |  |  |  | <.0001 |
| - No | 30663 (68.5%) | 16062 (60.7%) | 14601 (79.8%) |  |
| - Yes | 14109 (31.5%) | 10414 (39.3%) | 3695 (20.2%) |  |
| Liver Disease |  |  |  | <.0001 |
| - No | 1636 (3.7%) | 1251 (4.7%) | 385 (2.1%) |  |
| - Yes | 43136 (96.3%) | 25225 (95.3%) | 17911 (97.9%) |  |
| Treated Hypertension |  |  |  | <.0001 |
| - No | 38966 (87.0%) | 21862 (82.6%) | 17104 (93.5%) |  |
| - Yes | 5806 (13.0%) | 4614 (17.4%) | 1192 (6.5%) |  |

**Table S9. Demographics in model validation data set.**

Note: Values expressed as n(%), mean±standard deviation or median (Q1,Q3)

Note: P-value comparisons across case_flg categories are based on Chi-square test (or Fisher's Exact test) for categorical variables; p-values for continuous variables are based on ANOVA or Wilcoxon (Normal Approximation)

| **variable label** | **Overall N=44772** | **AFIB N=26476** | **No-AFIB N=18296** | **P-value** |
| --- | --- | --- | --- | --- |
|  |  |  |  |  |
| AGE (yrs) | 66.26 ± 13.61 | 71.29 ± 12.12 | 59.00 ± 12.32 | <.0001 |
| AGE (yrs) |  |  |  | <.0001 |
| - 40-55 | 11165 (24.9%) | 3125 (11.8%) | 8040 (43.9%) |  |
| - 56-66 | 11151 (24.9%) | 5803 (21.9%) | 5348 (29.2%) |  |
| - 67-77 | 11802 (26.4%) | 8487 (32.1%) | 3315 (18.1%) |  |
| - >77 | 10654 (23.8%) | 9061 (34.2%) | 1593 (8.7%) |  |
| GENDER |  |  |  | <.0001 |
| - F | 25011 (55.9%) | 13482 (50.9%) | 11529 (63.0%) |  |
| - M | 19761 (44.1%) | 12994 (49.1%) | 6767 (37.0%) |  |
| RACE |  |  |  | <.0001 |
| - Black | 2835 (6.3%) | 1507 (5.7%) | 1328 (7.3%) |  |
| - Other | 5481 (12.2%) | 3211 (12.1%) | 2270 (12.4%) |  |
| - White | 36456 (81.4%) | 21758 (82.2%) | 14698 (80.3%) |  |
| ETHNICITY |  |  |  | <.0001 |
| - HISPANIC OR LATINO | 339 (0.8%) | 131 (0.5%) | 208 (1.1%) |  |
| - NOT HISPANIC OR LATINO | 23904 (53.4%) | 13515 (51.0%) | 10389 (56.8%) |  |
| - Unknown | 20529 (45.9%) | 12830 (48.5%) | 7699 (42.1%) |  |
| Insurance Type |  |  |  | <.0001 |
| - COMMERCIAL | 20307 (45.4%) | 11460 (43.3%) | 8847 (48.4%) |  |
| - MEDICAID | 6087 (13.6%) | 3981 (15.0%) | 2106 (11.5%) |  |
| - MEDICARE | 9243 (20.6%) | 7385 (27.9%) | 1858 (10.2%) |  |
| - OTHER/UNKNOWN | 9135 (20.4%) | 3650 (13.8%) | 5485 (30.0%) |  |

**Supplementary Methods**

**Data transformation**

Variables (Appendices 1 and 2) were imported into SAS 9.4. The lab data consisted of repeated lab measurements per subject. This data was sorted by lab date and the most recent lab value extracted by lab type and patient ID. The distributions of each of lab values were examined and values in excess of the median +/- 1.5 intra-quartile range were discarded to reduce the impact of outlying values on the resulting analyses. Distribution of lab values by type before and after trimming were visualized using boxplots.

These distributions were then used to extract maximum and minimum values for relevant lab values and merged back into the lab data set after transposing to achieve a flat file of one record per unique patient. The flat lab files were then merged with the medical history and demographic files to arrive at a pre-analytic file consisting of almost 63K records.

Continuous Lab values were converted to classification variables to reflect low, baseline, and high categories and missing lab values were classified as normal (since labs are often not ordered when no abnormality is suspected). All lab values were coded as, 0=Low, 1=Normal, 2= Baseline High, and 3=High.

These new variables were appended with a “c” to distinguish them from their continuous counterparts. Additional variables that represented disease ensembles were used to create (0,1) variables.

Missing data were treated as follows:

- Merging lab data with demographic, medical and comorbidity data naturally resulted in patients with missing lab values.
- Missing data patterns for variables of interest were generated and examined using PROC MI (Multiple imputation)
- Missingness in the lab data were handled by assuming that a lab test was not ordered because it was not medically necessary and so it was decided to replace all missing lab values as normal
- IF missingness was excessive for any covariate / co-morbidity we constructed a separate class within the respective category.
- If missingness was slight and felt to not impact the results, those variables were left as is and left for list-wise deletion by SAS during the analyses.

**Statistical Analyses**

**Main statistical methods**

Our exploration of the data began using basic summary measures appropriate for the distribution of the respective variable(s). Chi-square tests for categorical variables and t-tests or non-parametric alternatives for continuous variables. Variables with p-values greater than 0.05, or whose sample contributed less than 1% to the data were discarded and were not included for secondary screening. Penalized stepwise Logistic Regression models using Schwartz-Bayes Criterion (SBC) were applied to all available variables that formed a union of the available literature-based risk models. The set of significant variables from this secondary screening were then passed through a best subsets macro that were ranked by AUC. A one percent difference from the maximal model was referenced as our baseline / parsimonious model and a more complex model subset was also referenced were any particular subset first reached the C-statistic of the maximal model. These initial models were highlighted and noted. This literature variable set was then allowed to interact in a pairwise fashion and again ranked by AUC noting only interactions that improved the AUC over a simpler main effects model.

All additional variables from our data extraction, and not part of the literature-based risk sets, were ran through a Random Forest procedure and only those variables with a positive out of bag Gini (Gini OOB) were selected as potential additions to the literature set and, as before, allowed to interact in a pairwise fashion and ranked by AUC. To test the robustness of the model selection procedure we created a “black box” set that consisted of every variable we had available plus combined disease (0,1) ensembles, and all pairwise interactions, were ran through the same Penalized Logistic regression procedure and again ranked by AUC and compared to the previous model runs.

Three models emerged consistently from the penalized Logistic regression model runs. The first was the most parsimonious model that differed from the maximal model by 1% in AUC, secondly the subset that first reached the C-statistic of the maximal set, and finally the model set from the penalized Logistic regression black box run consisting of all available variables that first reached the C-statistic of the maximal “black box” model run. These models plus the risk models from the literature were compared visually and analytically using their AUC curves and pairwise differences of the model(s) C-statistic(s). These selected models were then validated in an entirely different data set and their performance compared visually and analytically as previously discussed. To aid in the choice of models we listed all best subsets ranked by AUC but since we have multiple models with similar AUC’s we chose an example model to balance clinical relevance, ease of use in the EHR system, and parsimony. This Logistic model was run to output the risk probabilities. These were then used to find the optimal probability-based risk cut-offs using, (i) Youden’s Index, (ii) ER criterion (closest to 0,1), (iii) Concordance criterion (CZ), and (iv) Intersection union method (IU) to extract sensitivity and specificity. As an example of model performance these cutoffs were used, alongside a range of disease prevalences, in a final logistic regression model to create a table of sensitivity and specificities across these ranges. All analyses were performed using SAS 9.4.

### Sensitivity analyses

To test the sensitivity of the final model to data trimming we utilized a dataset trimmed by removing the upper and lower five percent of the data and output the AUC curves and compared to the Interquartile range method of trimming where we removed values plus or minus 1.5 interquartile ranges from the median. Sensitivity of model selection by method was performed using unpenalized Logistic stepwise Regression. The models selected were identical with no change in AUC. We also ran the variables through an ad-hoc informal data mining procedure utilizing LASSO, Least Angle Regression, and Random Forest procedures and found no significant differences in the variable lists produced in relation to those from utilizing our main penalized Logistic regression modeling approach.

We also ran the models within the validation set but excluded unstructured data. This was accomplished by referencing a text flag whose value was “1” if the data value came from an unstructured text search and “0” if not. If the variable had an associated text flag =1 and the variable itself =1, it was recoded as zero and created a new model variable appended with _Diag to indicate this is a pure diagnoses code variable. The resulting AUC for the UNAFIED model without NLP was 0.805.

**TRIPOD Statement**

| **Section/Topic** | **Item** |  | **Checklist Item** | **Section(s)/**  **Paragraph(s)** |
| --- | --- | --- | --- | --- |
| **Title and abstract** | | | | |
| Title | 1 | D;V | Identify the study as developing and/or validating a multivariable prediction model, the target population, and the outcome to be predicted. | Title |
| Abstract | 2 | D;V | Provide a summary of objectives, study design, setting, participants, sample size, predictors, outcome, statistical analysis, results, and conclusions. | Abstract |
| **Introduction** | | | | |
| Background and objectives | 3a | D;V | Explain the medical context (including whether diagnostic or prognostic) and rationale for developing or validating the multivariable prediction model, including references to existing models. | Background paragraphs 1,2,3 |
|  | 3b | D;V | Specify the objectives, including whether the study describes the development or validation of the model or both. | Background  paragraph 4 |
| **Methods** | | | | |
| Source of data | 4a | D;V | Describe the study design or source of data (e.g., randomized trial, cohort, or registry data), separately for the development and validation data sets, if applicable. | Study compliance & data sources and study design |
|  | 4b | D;V | Specify the key study dates, including start of accrual; end of accrual; and, if applicable, end of follow-up. | Study design and subjects |
| Participants | 5a | D;V | Specify key elements of the study setting (e.g., primary care, secondary care, general population) including number and location of centres. | Study setting |
|  | 5b | D;V | Describe eligibility criteria for participants. | Identification of cases and identification of controls |
|  | 5c | D;V | Give details of treatments received, if relevant. | N/A |
| Outcome | 6a | D;V | Clearly define the outcome that is predicted by the prediction model, including how and when assessed. | Study design and identification of cases |
|  | 6b | D;V | Report any actions to blind assessment of the outcome to be predicted. | N/A |
| Predictors | 7a | D;V | Clearly define all predictors used in developing or validating the multivariable prediction model, including how and when they were measured. | Candidate variable selection and supplementary table 1 |
|  | 7b | D;V | Report any actions to blind assessment of predictors for the outcome and other predictors. | N/A |
| Sample size | 8 | D;V | Explain how the study size was arrived at. | Identification of cases and identification of controls |
| Missing data | 9 | D;V | Describe how missing data were handled (e.g., complete-case analysis, single imputation, multiple imputation) with details of any imputation method. | Candidate variable selection |
| Statistical analysis methods | 10a | D | Describe how predictors were handled in the analyses. | Candidate variable selection and supplementary table 2 |
|  | 10b | D | Specify type of model, all model-building procedures (including any predictor selection), and method for internal validation. | Statistical methods for model development and validation |
|  | 10c | V | For validation, describe how the predictions were calculated. | Statistical methods for model development and validation |
|  | 10d | D;V | Specify all measures used to assess model performance and, if relevant, to compare multiple models. | Statistical methods for model development and validation |
|  | 10e | V | Describe any model updating (e.g., recalibration) arising from the validation, if done. | N/A |
| Risk groups | 11 | D;V | Provide details on how risk groups were created, if done. | N/A |
| Development vs. validation | 12 | V | For validation, identify any differences from the development data in setting, eligibility criteria, outcome, and predictors. | Study design and subjects |
| **Results** | | | | |
| Participants | 13a | D;V | Describe the flow of participants through the study, including the number of participants with and without the outcome and, if applicable, a summary of the follow-up time. A diagram may be helpful. | Results paragraph 1 and figure 1 |
|  | 13b | D;V | Describe the characteristics of the participants (basic demographics, clinical features, available predictors), including the number of participants with missing data for predictors and outcome. | Results paragraph 1 and table 1 |
|  | 13c | V | For validation, show a comparison with the development data of the distribution of important variables (demographics, predictors and outcome). | Supplementary table 9 |
| Model development | 14a | D | Specify the number of participants and outcome events in each analysis. | Results paragraph 1 and table 1 |
|  | 14b | D | If done, report the unadjusted association between each candidate predictor and outcome. | Table 1 and supplementary tables 3,4,7,8,9 |
| Model specification | 15a | D | Present the full prediction model to allow predictions for individuals (i.e., all regression coefficients, and model intercept or baseline survival at a given time point). | Table 2 |
|  | 15b | D | Explain how to the use the prediction model. | Table 2 |
| Model performance | 16 | D;V | Report performance measures (with CIs) for the prediction model. | Results paragraph 2 |
| Model-updating | 17 | V | If done, report the results from any model updating (i.e., model specification, model performance). | N/A |
| **Discussion** | | | | |
| Limitations | 18 | D;V | Discuss any limitations of the study (such as nonrepresentative sample, few events per predictor, missing data). | Discussion paragraph 7 |
| Interpretation | 19a | V | For validation, discuss the results with reference to performance in the development data, and any other validation data. | Discussion paragraph 1 |
|  | 19b | D;V | Give an overall interpretation of the results, considering objectives, limitations, results from similar studies, and other relevant evidence. | Discussion paragraphs 1-5 |
| Implications | 20 | D;V | Discuss the potential clinical use of the model and implications for future research. | Discussion paragraphs 6,8 |
| **Other information** | | | | |
| Supplementary information | 21 | D;V | Provide information about the availability of supplementary resources, such as study protocol, Web calculator, and data sets. | Additional file |
| Funding | 22 | D;V | Give the source of funding and the role of the funders for the present study. | Declarations/  Funding |

*Items relevant only to the development of a prediction model are denoted by D, items relating solely to a validation of a prediction model are denoted by V, and items relating to both are denoted D;V. We recommend using the TRIPOD Checklist in conjunction with the TRIPOD Explanation and Elaboration document.
